# Nanomedicine-powered redox homeostasis modulation: Disrupting antioxidant systems and inducing oxidative stress for precision tumor therapy

**DOI:** 10.1016/j.mtbio.2026.103315

**Published:** 2026-06-03

**Authors:** Jiale Zhan, Ruie Chen, Muhe Chen, Shuyue Yan, Yitian Zhang, Jiawen He, Ya Meng, Xiangrong Yu, Liewei Wen

**Affiliations:** a Guangdong Provincial Key Laboratory of Tumor Interventional Diagnosis and Treatment, Zhuhai People's Hospital (The Affiliated Hospital of Beijing Institute of Technology), Beijing Institute of Technology, Zhuhai, 519088, China; b Department of Radiology, Zhuhai Clinical Medical College of Jinan University (Zhuhai People's Hospital, the Affiliated Hospital of Beijing Institute of Technology), Zhuhai, 519000, China

**Keywords:** Redox balance, Oxidative stress, Antioxidant systems, Tumor therapy, Nanomedicine

## Abstract

Precise regulation of cellular redox balance is fundamental for maintaining normal physiological function and overall homeostasis. Oxidative stress arises when reactive oxygen species (ROS) overwhelm cellular antioxidant defenses, disrupting redox equilibrium. It plays a pivotal role in tumor initiation, progression, and metastasis. Tumor cells maintain remarkably elevated levels of both oxidants and antioxidants compared with normal cells, resulting in a dysregulated redox homeostasis that is particularly vulnerable to oxidative stress induced by exogenous stimuli. A growing body of evidence suggests that deliberate modulation of redox homeostasis through inducing intracellular ROS burst and disrupting antioxidant defense systems to enhance oxidative stress, represents a promising therapeutic strategy for precision tumor therapy. To date, various nanoparticles have been found to disrupt redox balance based on their catalytic activity, biological regulatory functions, and drug-delivery capabilities. In this review, we systematically overview oxidative and antioxidant systems in tumor cells and summarize recent advances in nanomedicine-based strategies for redox homeostasis modulation, with an emphasis on the regulation of metabolites, enzyme activities, and signaling pathways. Moreover, we evaluate current challenges and propose actionable strategies to promote clinical translation. By integrating mechanistic understanding with rational nanodrugs design, this review aims to support the development of redox-targeted therapeutic approaches and promote their future implementation in precision tumor therapy.

## Graphical abstract

**Figure:**
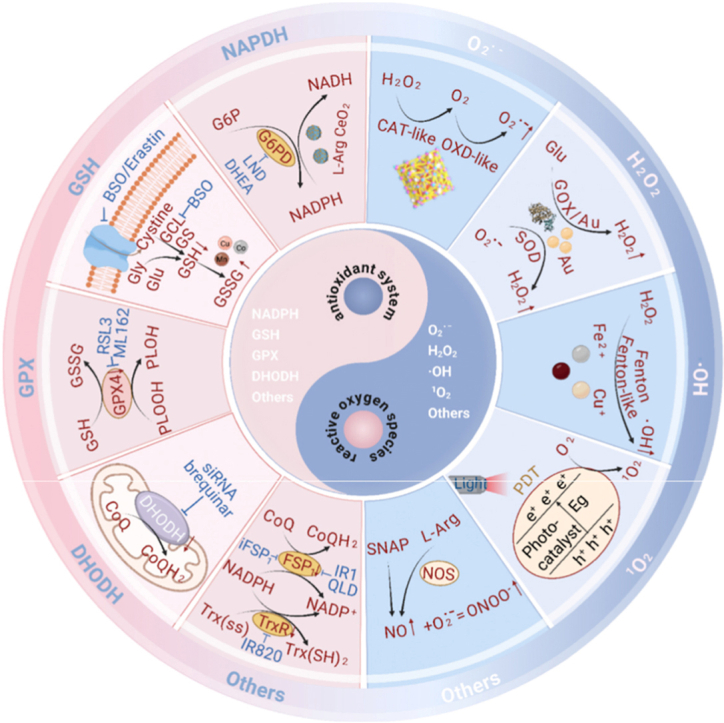

## 1 Introduction

Redox reactions are fundamental biochemical processes involving electron transfer between reducing and oxidizing agents that support cellular energy production, metabolism, and signal transduction in living systems [1]. The paradigm of biological redox activity is cellular respiration, wherein reduced cofactors such as NADH and FADH_2_ are oxidized through a series of enzymatic reactions in mitochondria [2]. The liberated electrons traverse the electron transport chain and ultimately reduce molecular oxygen (O_2_) to water. The energy released during this process drives proton translocation across the inner mitochondrial membrane, generating the proton motive force that powers the synthesis of adenosine triphosphate (ATP), the universal energy currency of life [3]. During normal aerobic metabolism, reactive intermediates known as ROS are generated and can serve as signaling molecules that regulate key cellular processes [4]. However, the excessive accumulation of ROS can directly cause severe structural and functional damage to vital biomacromolecules, including lipids, proteins, and nucleic acids [5]. To counteract this ROS overload, cells have evolved sophisticated antioxidant defense networks comprising both locally upregulated antioxidant pathways and elevated levels of antioxidant molecules [6]. The dynamic balance between oxidative and antioxidant systems establishes cellular redox homeostasis, which is essential for maintaining normal cellular function and enabling adaptive responses to environmental and pathological stimuli.

**Fig. 1:**
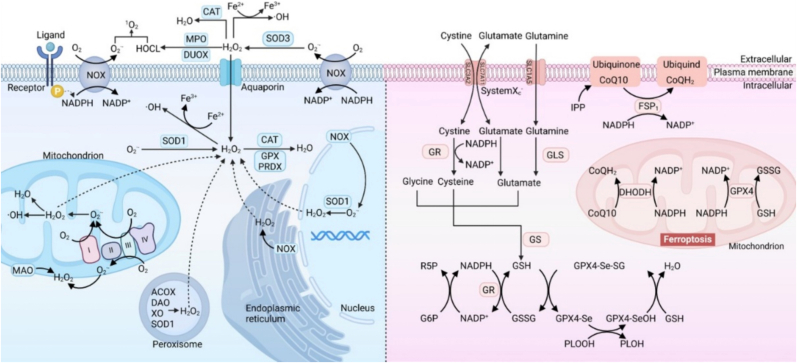
Intracellular redox balance and regulation. Oxidative processes are indicated in dark blue, while antioxidant processes are depicted in red. Created with Biorender (Agreement number: JE29P71VY2). (For interpretation of the references to color in this figure legend, the reader is referred to the Web version of this article.)

Oxidative stress, a concept first proposed by H. Sies in 1984, refers to a state in which the generation of ROS, including superoxide anion (O_2_·^-^), hydroxyl radical (·OH), hydrogen peroxide (H_2_O_2_), and singlet oxygen (^1^O_2_) overwhelms the capacity of cellular antioxidant defense systems [7,8]. Persistent oxidative stress causes oxidative damage to biomacromolecules and is closely associated with the pathogenesis and progression of multiple disease states, including aging, inflammatory disorders, neurodegenerative diseases, and tumors [9]. Particularlly, tumor cells intrinsically exist in a state of elevated oxidative stress owing to their distinct pathophysiological features, including metabolic reprogramming, mitochondrial dysfunction, sustained oncogenic signaling, and a tumor microenvironment characterized by hypoxia, inflammation, and nutrient deprivation [[10], [11], [12]]. Consequently, moderate accumulation of endogenous ROS accumulation not only promotes genomic instability and metabolic adaptation but also acts as secondary messenger to activate oncogenic signaling pathways that promote tumor proliferation, angiogenesis, and metastasis [13]. Meanwhile, to evade the lethal effects of this persistent oxidative burden, tumor cells concomitantly upregulate robust antioxidant defense systems, such as glutathione (GSH), thioredoxin (Trx), glutathione peroxidases (GPXs), and other related antioxidant enzymes, to continuously scavenge excess ROS (Fig. 1) [14]. This concurrent upregulation of both ROS generation and antioxidant scavenging establishes a robust, high-capacity dynamic equilibrium that maintains redox homeostasis.

Although this adaptive redox reprogramming supports tumor survival and progression, it also perilously places tumor cells in a state close to the threshold of oxidative tolerance, rendering them exceptionally vulnerable to further ROS elevation or disruption of antioxidant defenses [15]. Once the oxidative threshold is breached, severe oxidative damage to biomacromolecules can initiate multiple forms of regulated cell death. For instance, excessive ROS can induce apoptosis via mitochondrial cytochrome *c* release and subsequent caspase activation, or through severe endoplasmic reticulum (ER) stress [16]. In addition, ROS-driven mitochondrial DNA oxidation can trigger inflammasome activation and subsequent Gasdermin-mediated membrane pore formation, resulting in pyroptosis [17]. Furthermore, ROS generated through Fenton reactions can drive iron-dependent lipid peroxidation, while inhibition of the cystine/glutamate antiporter system Xc^−^ or direct inactivation of GPX4 disrupts redox homeostasis, resulting in uncontrolled lipid damage and ferroptosis [18]. Collectively, the intrinsic dependence of tumor cells on tightly regulated redox homeostasis provides a unique therapeutic window for selectively induce tumor cell death through amplifying oxidative stress or impairing antioxidant systems. Therefore, it is imperative to further elucidate the interplay between oxidative stress and tumor biology, including the factors driving oxidative stress and its impact on tumor progression, to develop more effective antitumor strategies.

**Fig. 2:**
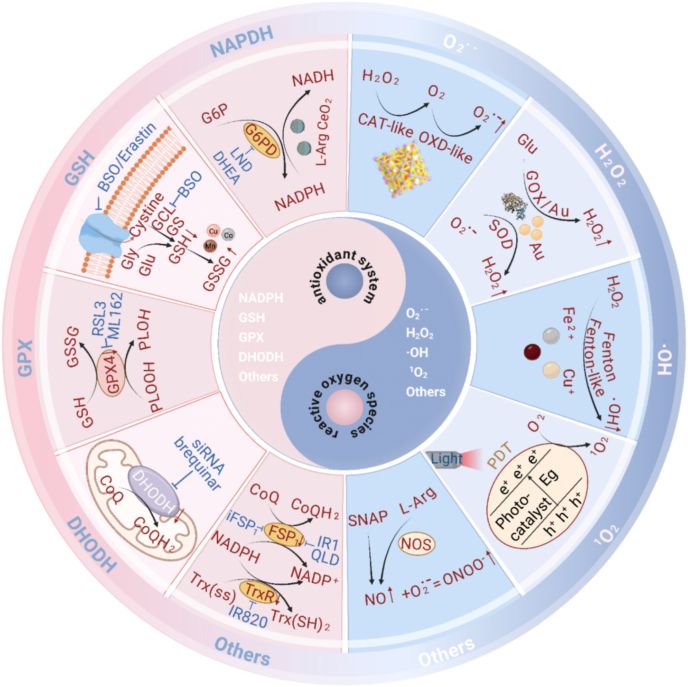
Nanomedicine-based strategies for tumor therapy through inducing intracellular ROS burst and disrupting antioxidant defense systems. Created with Biorender (Agreement number: TO29P6X476).

Over the past decades, extensive efforts have been devoted to developing nanomedicine-based tumor therapeutics focused on modulating redox homeostasis through two complementary mechanisms: inducing intracellular ROS burst and disrupting antioxidant defense systems [19]. The first strategy aims to amplify oxidative stress beyond the tolerance threshold of tumor cells through excessive ROS generation [20]. For example, redox-active nanomaterials or nanozymes can catalyze intracellular redox reactions to continuously generate O_2_·^-^ and H_2_O_2_, while transition-metal-based nanocatalysts promote Fenton or Fenton-like reactions to convert endogenous H_2_O_2_ into highly reactive ·OH [21,22]. In parallel, photodynamic therapy (PDT), sonodynamic therapy (SDT), and microwave dynamic therapy employ external energy stimulation to activate photosensitizers or sonosensitizers for localized ^1^O_2_ production [23]. Furthermore, extending beyond traditional ROS, the delivery of nitric oxide (NO) donors facilitates the reaction between NO and O_2_·^-^ to produce highly reactive peroxynitrite (ONOO^−^), triggering a devastating reactive nitrogen species (RNS) burst that further exacerbates oxidative stress [24]. It is worth noting that solely increasing ROS production is often compromised by the tumor's robust scavenging capacity. Thus, disrupting tumor's antioxidant defense systems has emerged as an equally important and complementary therapeutic strategy [25]. Representative approaches include depletion of NADPH, exhaustion of intracellular GSH, inhibition of GSH biosynthesis, and suppression of key antioxidant enzymes and pathways, such as GPX4, dihydroorotate dehydrogenase (DHODH), ferroptosis suppressor protein 1 (FSP1), and the thioredoxin system [[26], [27], [28], [29]]. Accordingly, nanomedicines engineered to depleting GSH, blocking cystine uptake through system Xc^−^ inhibition, or selectively inactivating antioxidant enzymes have demonstrated remarkable potential in triggering lipid peroxidation, ferroptosis, apoptosis, and other forms of ROS-dependent regulated cell death (Fig. 2) [18].

By systematically integrating these two fundamental pathways either independently or synergistically, nanomedicine can thoroughly disrupt the tumor's unique redox equilibrium [30]. This dual-modulation paradigm has birthed a diverse array of innovative nanotherapeutic paradigms and established a promising concept for presion tumor therapeutic strategy based on redox homeostasis modulation. In this review, we systematically summarize the oxidative and antioxidant networks in tumor cells and discuss recent advances in nanomedicine-based strategies for redox regulation, with particular emphasis on ROS amplification, antioxidant suppression, and their underlying molecular mechanisms. We further highlight current challenges and future opportunities in this rapidly evolving field, aiming to provide insights for the rational design and clinical translation of redox-targeted nanomedicines for precision oncology.

## 2 Inducing the burst of cellular ROS

ROS are central mediators of oxidative stress-induced tumor cell death [31]. Although moderate ROS levels are required for tumor-associated signaling and metabolic adaptation, excessive ROS accumulation beyond the antioxidant buffering threshold causes irreversible oxidative damage to biomacromolecules and subsequently triggers apoptosis, ferroptosis, pyroptosis, and other forms of regulated cell death [32,33]. Accordingly, deliberate amplification of intracellular ROS has emerged as one of the most extensively investigated strategies for redox-based tumor therapy [34]. Recent advances in nanomedicines have enabled efficient and controllable ROS generation within tumors [35]. Compared with conventional small-molecule therapeutics, nanodrugs possess unique advantages, including catalytic activity, tumor-targeting capability, stimulus responsiveness, and multifunctional integration, allowing spatiotemporally controlled amplification of oxidative stress in the tumor microenvironment [36]. Nanoplatforms can preferentially accumulate in tumors through the enhanced permeability and retention (EPR) effect or ligand-mediated active targeting, thereby improving therapeutic selectivity while reducing damage to normal tissues [37]. In addition, tunable physicochemical properties, including particle size, surface modification, and structural composition, facilitate prolonged circulation, enhanced tumor retention, and improved intracellular delivery [38]. More importantly, nanomedicines can integrate multiple redox-regulatory functions, such as in situ ROS generation, oxygen self-supply, GSH depletion, and cascade catalytic amplification, thereby synergistically disrupting tumor redox homeostasis [39].

Representative strategies include nanozyme-catalyzed redox reactions for continuous generation of O_2_·^-^ and H_2_O_2_, chemodynamic therapy (CDT) based on Fenton or Fenton-like reactions for ·OH production, and PDT utilizing photosensitizers to generate ^1^O_2_ under external stimulation. In addition, emerging approaches based on RNS, particularly NO-mediated oxidative damage, have also demonstrated considerable potential for tumor therapy (Table 1) [56].

**Table 1:** Overview of nanomedicine-based strategies for inducing cellular ROS burst.

Target	Intervention strategies	Representative examples	Advantages	Limitations	Ref.
O_2_·^-^	Nanozyme catalysis	Co-SAs@NC	Cascade catalysis; TME responsiveness	H_2_O_2_ dependence; Limited catalytic specificity	[40]
	PDT activation (Type I)	DA	Hypoxia tolerance; Efficient electron transfer	Poor tissue penetration; Light dependence	[41]
H_2_O_2_	GOx delivery	LMGC	High selectivity; Biodegradability	Glucose dependence; Enzyme instability	[42]
		PEG-GOx	Stability; Long circulation, Simple synthesis	pH dependence	[43]
	Metal-nanozyme catalysis	DMSN-Au-Fe_3_O_4_	High catalytic efficiency; H_2_O_2_ self-supply	Lower selectivity, Potential toxicity, Slower clearance	[44]
	Fenton conversion	PZIF67-AT	ROS cascade reactions; Multi-functional design	Complicate translation	[45]
·OH	CDT	MOF/TA-CDDP-Apt	Homologous targeting; Immune activation	Complex preparation	[46]
	Ferroptosis	Fe-DMOS@CaO_2_-HA NCs	Ferroptosis induction; High loading capacity	Low catalytic efficiency; Potential toxicity	[47]
	Pyroptosis	PEG-CuP-COF	Active targeting; Deep penetration	Potential infection; Complex preparation	[48]
^1^O_2_	PDT activation (Type II)	Porphyrins/chlorins-based nanomedicines	High ROS yield	Poor hydrophilicity; Oxygen-dependent	[49]
	Russell mechanism	Fe_3_O_4_-Au JNPs	Avoids light and radicals; Theranostics	Structural instability	[50]
	PDT + Hypoxia alleviation	HA-CAT@aCe6	Hypoxia alleviation; Low dark toxicity	H_2_O_2_ dependence; Light limitation	[51]
	PDT + LPO amplification	MAR	Ferroptosis propagation; Sustained ICD	Potential toxicity	[52]
Others	·NO induction	JS-K	Synergistic induction of apoptosis and autophagy	TME-independent	[53]
	NO delivery	T-SPM_DCTBT/NO_	Deep tissue penetration; ROS/RNS synergy	Difficult synthesis	[54]
	ONOO^−^ nanogenerator	APAP-P-NO	Tumor specificity; Immune activation	Potential toxicity	[55]

### 2.1 Superoxide radical (O_2_·^-^)

**Fig. 3:**
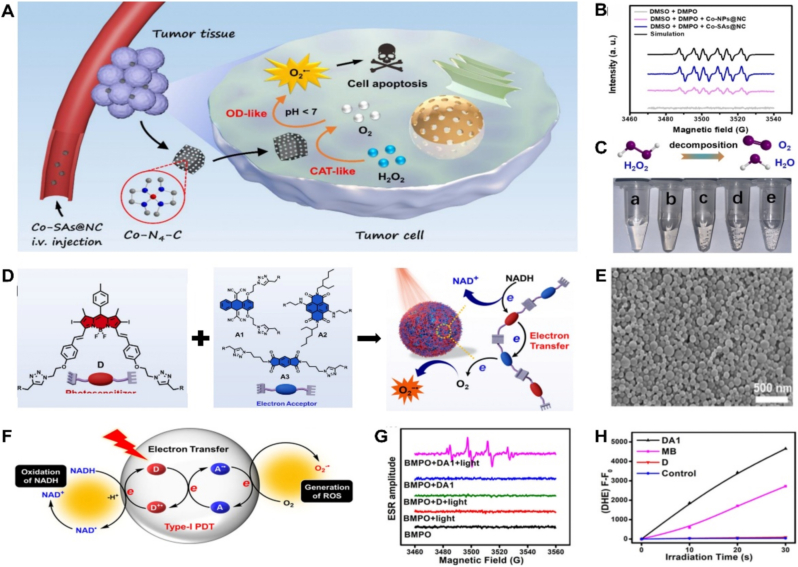
Nanomedicine-based strategies for O_2_·^-^ generation through enzyme-mimicking catalysis and photosensitization. **A)** Co-SAs@NC exhibits CAT-like and OXD-like properties. **B)** ESR spectra of O_2_·^-^ trapped by DMPO. **C)** The reaction scheme for H_2_O_2_ decomposition and photographs of O_2_ production in sodium acetate buffers (pH 6.0) containing different components. **D)** Schematic representation of PDT based on supramolecular photodynamic agents. **E)** SEM image of DA1. **F)** Schematic illustration of the photoinduced oxidation of NADH and generation of O_2_·^-^. **G)** ESR spectra to detect O_2_·^-^ generated by DA1 under illumination, using BMPO as a spin-trap agent. **H)** Plots of ΔFl. (F-F0) of DHE at 580 nm upon light irradiation for different time intervals in the presence of DA1 (a photodynamic agent), MB, or D (a classical Type-II PS, iodide-BODIPY). **A-C)** Reproduced with permission [40]. Copyright 2022, Elsevier Ltd. **D-H)** Reproduced with permission [41]. Copyright 2022, Springer Nature.

O_2_·^-^ is a reactive oxygen radical primarily generated through electron leakage from the mitochondrial electron transport chain, as well as via enzymatic reactions such as NADPH oxidase activity [2]. Compared with other ROS, O_2_·^-^ exhibits moderate reactivity and carries a negative charge, which restricts its membrane permeability and confines its activity to the site of generation [57]. It serves as a primary ROS species and a key precursor in the cascade of ROS formation. In recent decades, various methods have been devised to increase introtumoral O_2_·^-^ levels for tumor therapy, such as nanozyme-dependent strategy and PDT [58]. Nanozymes, synthetic nanomaterials with enzyme-mimetic catalytic activity, have attracted intensive interest owing to their tunable catalysis, excellent chemical and thermal stability, and low cost for large-scale production [59,60]. Nanozymes typically exhibit multiple enzyme-mimicking activities, such as peroxidase (POD)-like and oxidase (OXD)-like activities, enabling the conversion of endogenous H_2_O_2_, which has relatively low reactivity, into more reactive and cytotoxic ROS, such as O_2_·^-^ and ·OH, thereby amplifying oxidative stress [61]. For instance, a recent study by Cai et al. developed a single-atom nanozyme (Co-SAs@NC) featuring atomically dispersed cobalt centers coordinated with nitrogen within a porous carbon matrix. This nanoplatform elegantly integrated dual enzyme-mimetic activities to drive a catalytic cascade (Fig. 3A). Specifically, its catalase (CAT)-like activity decomposed endogenous H_2_O_2_ into O_2_, which was subsequently reduced to O_2_·^-^ via electron transfer driven by its OXD-like activity. Notably, this cascade was selectively activated under mildly acidic conditions (pH 6.0), generating abundant cytotoxic O_2_·^-^ to effectively trigger tumor cell apoptosis (Fig. 3B and C) [40].

Among the various ROS-generating strategies, PDT has emerged as one of the most extensively investigated and clinically translated approaches for inducing oxidative stress in tumors since the 1960s [62]. In typical PDT, photosensitizers (PSs) absorb specific wavelengths of light, and transition from the ground state to excited singlet or triplet state. In the presence of molecular oxygen, these excited PSs initiate photochemical reactions that selectively damage tumor tissues [63]. In particular, type-I photosensitization involves electron or charge transfer processes that generate ROS such as O_2_·^-^ and ·OH, thereby inducing oxidative destruction of tumor cells [64]. For example, Teng's group engineered a novel type-I photosensitizer (DA) through supramolecular self-assembly driven by quadruple hydrogen bonding (Fig. 3D and E). Under light irradiation, DA simultaneously produces highly oxidative cationic radicals and O_2_·^-^ (Fig. 3F and G), reaching a radical yield equivalent to 1.7 times that of conventional methylene blue (Fig. 3H) [41]. Owing to the potent oxidative activity and broad downstream reactivity of O_2_·^-^, such type-I PDT strategies provide a promising platform for nanocatalytic tumor therapy, particularly in hypoxic tumor microenvironments where conventional type-II PDT is often limited.

Despite above advances, O_2_·^-^-based tumor therapeutic strategy still face several intrinsic limitations. O_2_·^-^ possesses a short lifetime and limited diffusion distance, which restricts its effective cytotoxic range within tumors [65]. In addition, the efficiency of ROS production is often constrained by insufficient oxygen availability, limited endogenous substrates, and rapid antioxidant scavenging in the tumor microenvironment. For PDT, photobleaching, poor tissue penetration of excitation light, and instability of photosensitizers can further reduce sustained ROS generation and therapeutic efficacy [66]. Moreover, excessive reliance on a single ROS-generating mechanism may be insufficient to overcome the complex redox adaptation and heterogeneity of tumors. Therefore, current research increasingly focuses on developing multifunctional nanoplatforms capable of sustaining or amplifying O_2_·^-^ production through cascade catalytic reactions, self-supplying oxygen strategies, or continuous redox cycling [67]. Simultaneously, integrating with complementary modalities, including chemodynamic therapy, gas therapy, immunotherapy, ferroptosis induction, and antioxidant defense disruption has emerged as a promising direction for achieving more comprehensive and durable antitumor efficacy.

### 2.2 Hydrogen peroxide (H_2_O_2_)

Endogenous H_2_O_2_ is primarily generated from the dismutation of superoxide produced by the mitochondrial electron transport chain, as well as from various oxidase-mediated metabolic processes, including NADPH oxidases and xanthine oxidase, with additional contributions from peroxisomes and the endoplasmic reticulum [2,68]. Compared with other ROS, H_2_O_2_ is relatively stable and membrane-permeable, enabling its diffusion across cellular compartments and selective oxidation of target proteins, thereby achieving tumor-killing effects. In addition, endogenous H_2_O_2_ plays a key mediating role in redox signaling and ROS cascade reactions [69]. It can generate ·OH in cells through Fenton or Fenton-like reactions, which can be converted into highly toxic ·OH in cells through Fenton or Fenton-like reactions, further amplifying ROS generation, effectively disrupting tumor redox homeostasis, and inducing various programmed cell deaths in tumor cells [70]. Owing to its stability and convertibility, H_2_O_2_ has emerged as a crucial entry point for nanomedicine-based cancer therapy.

**Fig. 4:**
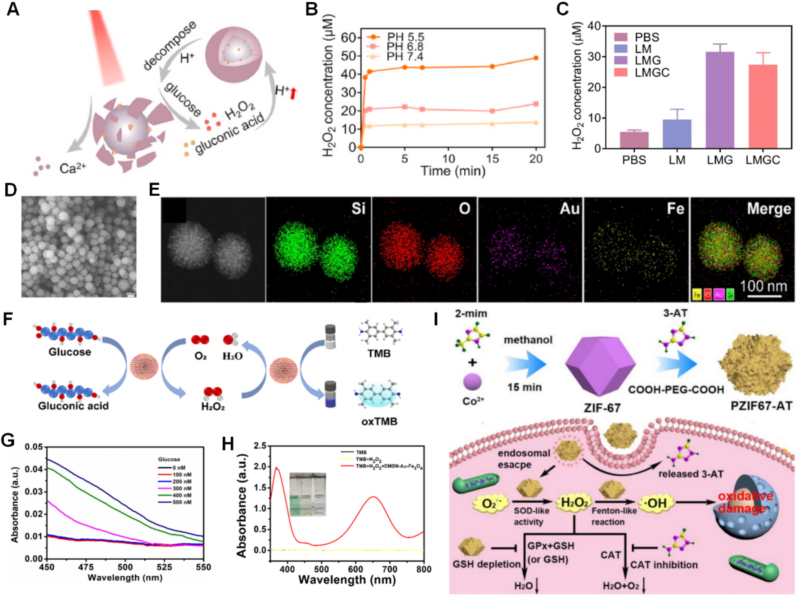
Increase intracellular H_2_O_2_ levels by promoting H_2_O_2_ production and inhibiting H_2_O_2_ elimination. **A)** The degradation process of LMGC in acidic environment. **B)** H_2_O_2_ generation in LMGC solution at different pH. **C)** Evaluated intracellular H_2_O_2_ concentration of CT26 cells after different treatment. **D)** SEM image of DMSN. **E)** Dark-field STEM image of DMSN-Au-Fe3O4 NPs and corresponding element mappings for elements of O, Si, Au, Fe, and merge, respectively. **F)** Schematic illustration of DMSN-Au-Fe_3_O_4_ nanoplatform-catalyzed glucose oxidation reaction. **G)** UV-vis absorption spectra of DMSN-Au-Fe_3_O_4_ after incubation with glucose at varied concentrations for 1 h. **H)** UV-vis absorption spectra of the catalyzed oxidation of TMB (oxTMB) in the different reaction system (pH = 6.5). **I)** Schematic representation of the use of PZIF67-AT nanoparticles to induce intensive CDT. **A-C)** Reproduced with permission [42]. Copyright 2022, Biomaterials. **D-H)** Reproduced with permission [44]. Copyright 2022, The Royal Society of Chemistry. **I)** Reproduced with permission [45]. Copyright 2020, ACS Publications.

Current therapeutic strategies primarily involve delivering or mimicking the activity of H_2_O_2_ generating enzymes (such as SOD, GOx, and NOX) to catalyze H_2_O_2_ production and induce tumor oxidative stress. For example, using CaCO_3_ nanoparticles to deliver GOx and eutectic alloy gallium-indium (called as LMGC) can significantly inhibit tumor growth by catalyzing the oxidative stress caused by GOx-catalyzed glucose-to-H_2_O_2_ production (Fig. 4A), in conjunction with the photothermal effect of eutectic alloy gallium-indium (Fig. 4B–C) [42]. Besides, Fu et al. developed a PEG-GOx-encapsulated and DOX-loaded CuCaP nanoplatform (PGC-DOX) using a facile one-step biomimetic mineralization strategy [43]. Within this system, glucose oxidase (GOx) catalyzes the oxidation of glucose into H_2_O_2_, simultaneously inducing nutrient deprivation in tumor cells and supplying endogenous H_2_O_2_ for subsequent Cu ^+^ -mediated Fenton-like reactions. Meanwhile, the released DOX further enhances oxidative stress and chemotherapeutic efficacy. Importantly, encapsulation of GOx within the nanostructure significantly improves its stability and prolongs its biological activity, thereby overcoming the short half-life, poor stability, and systemic toxicity commonly associated with free GOx administration. Compared with endogenous ROS-generating enzymes, certain transition metal ions, including Fe^2+^, Cu^+^, and Mn^2+^, can also exhibit intrinsic oxidase-like catalytic activity under specific conditions, enabling continuous ROS production for nanocatalytic tumor therapy. Building on these properties, nanozymes possess high catalytic activity, excellent stability, and tunable structures and functions, enabling them to not only mimic natural enzymes but also achieve multifunctional catalysis. As a result, they have been widely applied in the generation and regulation of ROS. Particularlly, metal-based nanozymes have emerged as highly attractive tools for redox regulation in recent years. Mao et al. employed Au nanoparticles (Fig. 4D and E) with intrinsic GOx-mimicking activity to elevate intracellular H_2_O_2_ (Fig. 4F). The generated H_2_O_2_ is subsequently transformed into highly reactive ·OH by the POD-mimicking activity of Fe_3_O_4_, via Fenton reactions (Fig. 4G–H) [44].

Another strategy is to promote H_2_O_2_ generation while inhibiting the activity of H_2_O_2_ scavenging systems (such as CAT and GPX), thereby synergistically inducing oxidative stress to kill tumors. For instance, Sang et al. developed the nanocatalyst PZIF67-AT, which disrupts H_2_O_2_ homeostasis by simultaneously enhancing H_2_O_2_ production and hindering its removal [45]. In this nanodrug, superoxide-dismutase-mimetic activity converts O_2_·^-^ to H_2_O, while concomitant inhibition of catalase and depletion of GSH slow the decomposition of H_2_O_2_ to H_2_O (Fig. 4I). The resulting H_2_O_2_ accumulation intensifies Fenton-type reactions, thereby inducing substantial tumor-cell death. In summary, the diverse sources of H_2_O_2_ and its central role in ROS cascade amplification make it an attractive target for redox regulation, and innovative materials and strategies for modulating intracellular H_2_O_2_ levels hold great promise in cancer therapy.

### 2.3 Hydroxyl radicals (·OH)

·OH are the most reactive and short-lived free radicals in the human body, with a lifespan of approximately 10^−9^ s [71]. These radicals can damage biological macromolecules, including lipids, proteins, nucleic acids, and enzymes, thereby disrupting cellular structure and function and ultimately leading to necrosis or mutation [[72], [73], [74]]. Highly reactive ·OH is primarily produced from H_2_O_2_ through Fenton or Fenton-like reactions mediated by Fe^2+^ under acidic conditions, enabling ·OH serve as a central effector that bridges chemodynamic therapy (CDT) chemistry with downstream biological responses [75]. In addition, ·OH can also be generated via external stimuli such as light irradiation, radiolysis, and electrochemical reactions. Owing to its extremely high oxidative potential, ·OH rapidly attacks nearby biomacromolecules, particularly polyunsaturated fatty acids (PUFAs) within cellular membranes, thereby initiating lipid peroxidation (LPO) cascades and ultimately inducing ferroptosis [76]. Beyond ferroptosis, excessive ·OH accumulation can also activate inflammatory cell-death pathways. It has been reported to promote caspase-1 activation and Gasdermin D (GSDMD)-mediated membrane pore formation, thereby inducing pyroptosis and facilitating the release of damage-associated molecular patterns (DAMPs) [77]. Consequently, ·OH has emerged as one of the most potent cytotoxic ROS species and a widely exploited mediator in redox-based tumor therapy.

**Fig. 5:**
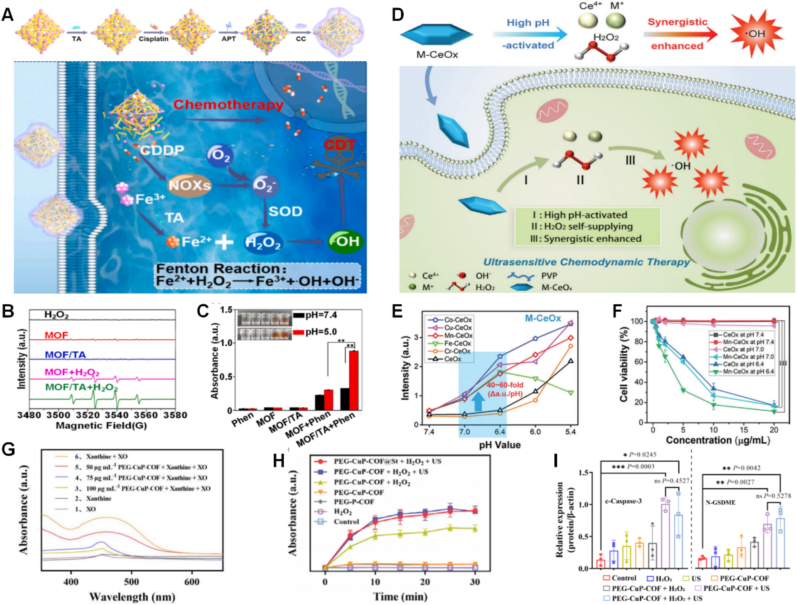
Tumor-targeted nanotherapeutic strategies that amplify ·OH production through Fenton and Fenton-like catalysis. **A)** Preparation of homologous-targeted hydroxyl radical nanogenerators and catalytic activity of M@MTCA in cells. **B)** ESR spectra of different samples with DMPO as the ·OH trap. **C)** Absorbance of PM, MOF, MOF/TA, MOF + PM, MOF/TA + PM under pH = 7.4 and pH = 5. **D)** Schematic design of Fenton-type bimetallic peroxides for tumor-specific catalytic therapy. **E)** TMB colorimetric detections of ·OH generation induced by M-CeOx. **F)** Effect of CeOx and Mn-CeOx on the viability of 4T1 cancer cells measured by MTT assay. **G)** SOD-mimicking activity. **H)** POD-mimicking activity of PEG-CuP-COF. **I)** Western blot quantitative analysis of expression levels of pyroptosis-related proteins (c-Caspase-3, N-GSDME) in RM-1 cells for various groups. **A-C)** Reproduced with permission [46]. Copyright 2021, Elsevier Ltd. **D-F)** Reproduced with permission [80]. Copyright 2021, John Wiley & Sons. **G-I)** Reproduced with permission [48]. Copyright 2024, Wiley-VCH.

In recent years, in situ Fenton and Fenton-like reactions triggered by metal ions such as iron (Fe), copper (Cu), manganese (Mn), vanadium (V) and their derivatives have emerged as one of the most widely used strategies in ·OH-dependent CDT [78,79]. Our group has proposed a synergistic CDT enhancement strategy based on a platinum and iron organic nanocomposite, MOF/TA-CDDP-Apt (M@MTCA) (Fig. 5A) [46]. Cisplatin (CDDP) elevates intracellular H_2_O_2_ through a cascade reaction. Simultaneously, Fe^2+^ reduced by tannic acid (TA) rapidly catalyzes the decomposition of hydrogen peroxide into ·OH (Fig. 5B and C). Consequently, M@MTCA synergistically maintains efficient ·OH production through the integration of platinum and iron, representing a promising tumor-targeted strategy for enhanced CDT. Besides, Zhou et al. developed the Fenton-type bimetallic peroxide for ultrasensitive CDT (Fig. 5D). This platform combines high-pH activation, metal-mediated synergy, and self-supplied H_2_O_2_ to drive cascade Fenton reactions [80]. Their bimetallic peroxides undergo ultrasensitive, acid-activated, decomposition-mediated Fenton-like reactions even at mildly acidic pH (6.5-7.0), significantly boosting ·OH production through synergistic metal catalysis and in situ H_2_O_2_ generation (Fig. 5E and F). Moreover, these bimetallic peroxides accumulate efficiently in tumors, rendering them potent catalytic therapeutic agents with negligible *in vivo* side effects.

·OH play critical roles in various biological processes, including the regulation of cell death pathways such as ferroptosis and pyroptosis. Ferroptosis is a form of regulated cell death characterized by the accumulation of ROS and enhanced LPO. LPO is typically initiated by hydrogen abstraction from methylene groups in polyunsaturated fatty acids (PUFAs), leading to the formation of lipid radicals. Due to its high reactivity, ·OH can efficiently initiate this process, promoting LPO accumulation and ultimately triggering ferroptosis in tumor cells [81]. For example, Fe^3+^-doped dendritic mesoporous organosilica nanoparticles (Fe-DMOS@CaO_2_-HA NCs) can be reduced to Fe^2+^ in the tumor microenvironment, thereby catalyzing Fenton reactions to generate abundant ·OH. Meanwhile, CaO_2_-mediated Ca^2+^ overload induces mitochondrial dysfunction, synergistically amplifying oxidative stress and lipid peroxidation [82,83]. Moreover, accumulating evidence indicates that ·OH, as one of the most reactive ROS, can effectively trigger pyroptosis by activating inflammasomes and inducing oxidative damage to mitochondria and cell membranes. A previous study demonstrates that multienzyme-mimicking covalent organic framework (PEG-CuP-COF) nanozymes act as specific pyroptosis inducers. These nanozymes exhibit both SOD- and POD-like activities, enabling the catalytic generation of H_2_O_2_ and ·OH (Fig. 5G and H), which subsequently activate the Caspase-3/Gasdermin E (GSDME)-dependent pyroptosis pathway to suppress tumor growth (Fig. 5I). Moreover, this nanozyme induces robust immune memory, effectively preventing bone metastasis [48].

In summary, ·OH possesses extremely high reactivity and represent one of the most potent cytotoxic ROS exploited in redox-based tumor therapy [84]. ·OH-mediated oxidative damage can efficiently amplify antitumor efficacy and overcome certain forms of therapeutic resistance, owing to the rapid oxidation of biomacromolecules, induction of lipid peroxidation, and disruption of organelle homeostasis. However, several critical challenges continue to limit the clinical translation of ·OH-dependent therapeutic strategy. First, the efficacy of Fenton and Fenton-like reactions is highly dependent on the availability of endogenous H_2_O_2_, which is often insufficient or heterogeneously distributed within tumors [84]. Although exogenous H_2_O_2_ supplementation or H_2_O_2_-generating systems can enhance ·OH production, excessive H_2_O_2_ accumulation may also induce nonspecific oxidative injury and raise biosafety concerns in normal tissues. Second, the ultrashort lifetime and restricted diffusion distance of ·OH confine its cytotoxic effects to regions immediately adjacent to the catalytic site, making efficient intracellular delivery and precise subcellular localization particularly important. In addition, tumor heterogeneity, fluctuating pH, hypoxia, and limited catalytic efficiency under physiological conditions further complicate the sustained generation of ·OH within the tumor microenvironment. Therefore, in addition to supplementting the substrate availability, and improving catalytic efficiency, precise spatiotemporal control over ·OH generation together with sustained disruption of tumor redox homeostasis will be critical for advancing ·OH-dependent nanomedicines toward clinically effective precision tumor therapy.

### 2.4 Singlet oxygen (^1^O_2_)

**Fig. 6:**
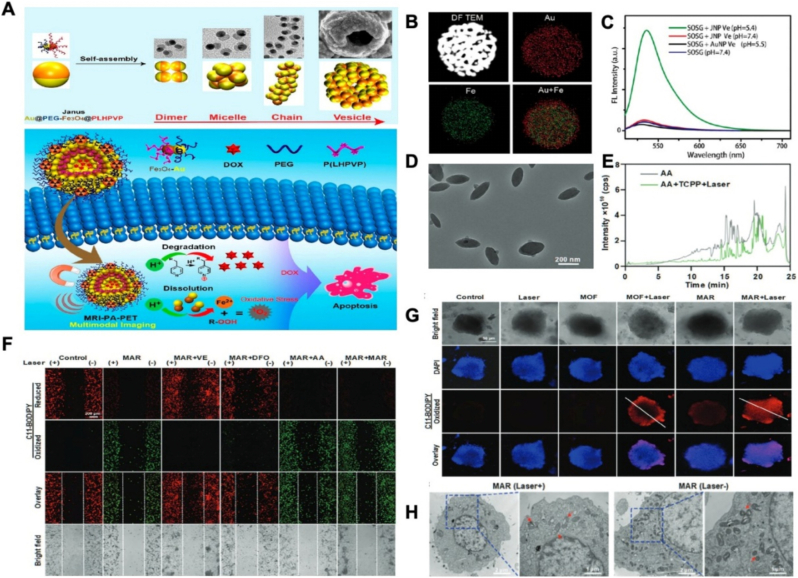
Nanomedicine-based strategies for ^1^O_2_ generation through Russell mechanism and photosensitizers. **A)** Schematic representation of the self-assembly of amphiphilic Janus NPs into dimer, micelle, chain, and bilayered vesicles and their corresponding simulation results and schematic illustration of the anticancer effect of doxorubicin (DOX)-loaded bilayered magnetic-plasmonic vesicles. **B)** Dark-field (DF) TEM and element mapping (Fe and Au elements) of a single vesicle. **C)** Fluorescence detection of ^1^O_2_ generation by ^1^O_2_ sensor green (SOSG). **D)** The TEM image of MOF NPs. **E)** Total ion current chromatographs of AA and AA + TCPP + Laser. **F)** Schematic diagram of the lineation method to divide the petri dishes into illuminated and non-illuminated regions and confocal microscope images of C11 BODIPY probe labeling CT26 cells for lipid peroxidation after the indicated treatments. **G)** CSLM images of C11 BODIPY probe labeling multicellular CT26 tumor spheroids under different treatments. **H)** Bio TEM images of CT26 cells treated with MAR NPs under laser irradiation and non-illuminated irradiation. **A-C)** Reproduced with permission [50]. Copyright 2019, ACS Publications. **D-H)** Reproduced with permission [52]. Copyright 2024, John Wiley & Sons. (For interpretation of the references to color in this figure legend, the reader is referred to the Web version of this article.)

As an electronically excited state of molecular oxygen, ^1^O_2_ is characterized by its elevated energy levels and potent electrophilicity. It can be generated through diverse pathways, including photocatalysis, chemical decomposition, and advanced oxidation processes [[85], [86], [87], [88]]. Extensive studies have demonstrated that photosensitizers such as porphyrins and chlorins can efficiently generate ^1^O_2_ upon light activation, thereby exerting PDT effects [49]. Beyond conventional PDT-based mechanisms, recent studies have revealed that certain non-photonic catalytic processes can also efficiently generate ^1^O_2_. In particular, the Russell mechanism has attracted increasing attention as an alternative pathway for singlet oxygen production under tumor-specific conditions. In this process, catalytic metal ions (such as Cu^2+^, Ce^4+^, and Fe^3+^) or specific enzymes promote the decomposition and recombination of lipid hydroperoxides or peroxyl radicals, ultimately yielding ^1^O_2_. Based on this concept, a variety of nanomaterials exploiting the Russell mechanism or related tumor-responsive catalytic reactions have recently been engineered for enhanced ^1^O_2_ generation [89,90]. For instance, Song et al. developed a magnetic-plasmonic bilayer vesicle capable of simultaneous ROS generation and DOX release. These double-layered vesicles self-assemble from amphiphilic iron-oxide-gold Janus nanoparticles (Fe_3_O_4_-Au JNPs) (Fig. 6A and B) [50]. In the acidic tumor microenvironment, Fe^2+^ liberated from the vesicles catalyzes substantial ^1^O_2_ formation through the Russell mechanism (Fig. 6C). The vesicles achieve superior inhibition of tumor growth and provide multimodal imaging, rendering this hybrid architecture a promising platform for tumor therapy.

As the most classical ^1^O_2_-based therapeutic modality, PDT has been widely applied in clinical tumor therapy due to its high spatiotemporal selectivity and minimal invasiveness. PDT relies on molecular oxygen as an essential substrate, in which light-excited photosensitizers transfer energy or electrons to O_2_ to generate ^1^O_2_ [91]. However, the therapeutic efficacy of PDT is often severely limited by the hypoxic nature of solid tumors, insufficient oxygen diffusion, and rapid oxygen consumption during photoreactions [92]. Therefore, inadequate intratumoral oxygen availability remains a major bottleneck restricting sustained ^1^O_2_ production and effective PDT outcomes. To overcome these limitations, extensive efforts have been devoted to alleviating tumor hypoxia and enhancing oxygen utilization efficiency through various strategies, such as hyperbaric oxygen therapy, hyperthermia, O_2_ delivery, artificial hemoglobin, oxygen-generating hydrogels, and peroxide-based materials [93]. For instance, the HA-CAT@aCe6 nanosystem effectively decomposes endogenous H_2_O_2_ via its encapsulated catalase to generate O_2_ in situ. This targeted oxygen evolution alleviates hypoxia within solid tumors, thereby potentiating ^1^O_2_ production and significantly enhancing the therapeutic efficacy of PDT [51].

It is worth noting that the therapeutic efficacy of PDT remains constrained by two intrinsic limitations: the shallow tissue penetration of excitation light and the restricted cytotoxic range of ^1^O_2_. To address these challenges, our team developed a GSH-activated nanophotosensitizer loaded with arachidonic acid (AA) and cloaked with a red-blood-cell membrane, functioning as a laser-triggered lipid-peroxidation nanoamplifier (MAR) (Fig. 6D and E) [52]. Upon laser irradiation, this platform enhances ROS generation, including ^1^O_2_, and drives a sustained lipid-peroxidation chain reaction, thereby amplifying ferroptosis in cancer cells. As a result, even deep-seated tumor cells that are not directly illuminated undergo ferroptosis owing to propagation of the death signal (Fig. 6F and G) [94]. Overall, this lipid peroxidation-propagating therapeutic strategy effectively amplifies oxidative stress in tumor cells, overcomes the limitation of the short lifetime of ^1^O_2_, expands the effective treatment range, and enhances post-treatment immune stimulation (Fig. 6H).

In summary, ^1^O_2_-dependent therapy, particularly PDT, has become one of the most extensively investigated and clinically translated ROS-based tumor therapeutic strategy. Although the conventional PDT still faces several intrinsic limitations, emerging non-photonic dynamic therapies, particularly SDT and microwave dynamic therapy (MWDT), have shown great promising in overcoming these barriers and attracted increasing attention in recent years [95,96].Compared with light-triggered PDT, ultrasound and microwave stimulation provide substantially deeper tissue penetration and broader treatment coverage, enabling more efficient ROS generation in deep-seated tumors [96]. In particular, nanoplatforms integrating sonosensitizers, microwave sensitizers, nanozymes, or oxygen-generating systems have demonstrated considerable potential for enhancing localized ^1^O_2_ production while simultaneously improving tumor penetration, catalytic efficiency, and therapeutic selectivity.

### 2.5 Others

In addition to the widely recognized ROS in tumor cells, other species, particularly RNS, also contribute to oxidative stress and tumor therapy. Key RNS include nitric oxide radical (·NO), ONOO^−^, and nitrogen oxides (NOx). Their primary source is the nitric-oxide-synthase-catalyzed conversion of L-arginine to ·NO, although ·NO can also arise via the nitrate-nitrite-nitric-oxide (NO_3_^−^, NO_2_^−^, NO) pathway. Intracellular ·NO readily reacts with existing ROS to form additional RNS. During the NOS-mediated conversion of L-arginine, O_2_·^-^ is produced, and ·NO rapidly combines with O_2_·^-^ to generate the highly reactive ONOO^−^, which can further react with ·NO to yield NO_2_ [97]. Thus, ONOO^−^ is considered an intermediate or donor of NO_2_ in these transformations. NO_2_ and ·NO can subsequently react to form species such as N_2_O_3_ [98]. Under physiological conditions, physiological concentrations of RNS act as signaling mediators, activating defense pathways that inhibit tumor initiation and protect normal cells. In the acidic, hypoxic tumor microenvironment (TME), however, ·NO reacts swiftly with O_2_·^-^, producing excess ONOO^−^. Indeed, tumor tissues contain higher levels of RNS than normal tissues [99]. When RNS generation surpasses the antioxidant capacity of tumor cells, growth inhibition or cell death can occur.

**Fig. 7:**
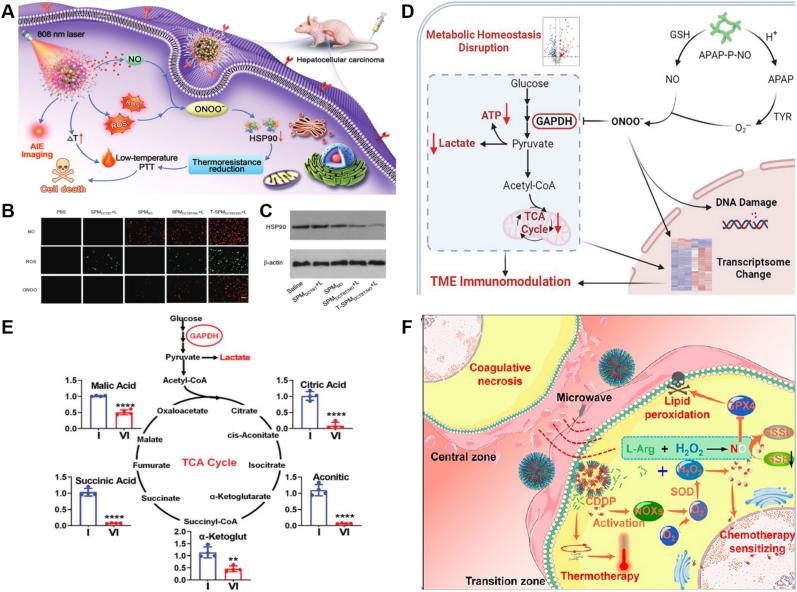
Nanoplatforms inducing reactive nitrogen species (RNS) generation and lipid peroxide accumulation for oxidative tumor therapy. **A)** Schematic illustration of the supramolecular nanocarrier T-SPM_DCTBT/NO_ for ONOŌ-potentiated mild-temperature PTT of hepatocellular carcinoma (HCC). **B)** Detection of intracellular NO, ROS, and ONOŌ by fluorescent microscopy after LM3 cells were treated with SPM_DCTBT_ plus laser irradiation (SPM_DCTBT_ + L), SPM_NO_, SPM_DCTBT/NO_ plus laser irradiation (SPM_DCTBT/NO_ + L), and T-SPM_DCTBT/NO_ plus laser irradiation (T-SPM_DCTBT/NO_ + L), respectively. **C)** The detection of intracellular HSP90 levels by Western blot assay after LM3 cells received different treatments. **D)** Schematic illustration of melanoma-specific ONOŌ overproduction-mediated metabolic homeostasis disruption for TME immunomodulation. **E)** Intracellular levels of TCA cycle metabolites measured by LC-MS/MS. **F)** Schematic Illustration of the Thermoacoustic Imaging-Guided Thermo-Chemotherapy with Nanocapsules (NPs@CDDP). **A-C)** Reproduced with permission [54]. Copyright 2023, John Wiley & Sons. **D-E)** Reproduced with permission [55]. Copyright 2023, John Wiley & Sons. **F)** Reproduced with permission [101]. Copyright 2023, ACS Publications.

Increasing evidence shows that ·NO donors can serve as targeted agents to reverse chemotherapy and immunotherapy resistance, thereby exerting potent antitumor effects [100,101]. For instance, the ·NO donor JS-K, investigated as a novel treatment for ovarian cancer, suppresses cell proliferation, induces nuclear condensation, and triggers apoptosis [53]. Numerous nanotherapies that harness RNS chemistry show considerable promise for cancer treatment. Hu et al. designed a supramolecular drug nanocarrier that co-delivers NO and the photothermal agent DCTBT for room-temperature photothermal therapy (PTT) (Fig. 7A) [54]. Under reductive intracellular conditions, the carrier releases NO efficiently, and irradiation with an 808 nm laser prompts DCTBT to generate both ROS and local hyperthermia. The resulting ROS react with NO to yield ONOO^−^, a potent oxidizing and nitrating agent (Fig. 7B). ONOO^−^ suppresses heat-shock-protein expression (Fig. 7C), lowers cancer-cell thermotolerance, and thereby achieves potent antitumor activity at mild temperatures (<50 °C).

Beyond their direct cytotoxicity, RNS can disrupt tumor-cell metabolic homeostasis and amplify antitumor immunity [54]. Lijun Yang and co-workers constructed a tumor-specific ONOO^−^ nanogenerator, APAP-P-NO, that selectively upsets metabolism in melanoma cells (Fig. 7D). Within the acidic, glutathione-rich, tyrosinase-active milieu characteristic of melanoma, APAP-P-NO produces O_2_·^-^ and liberates NO, which couple in situ to form ONOO^−^. Metabolomic analysis revealed that the accumulation of ONOO^−^ markedly depleted intermediates of the TCA cycle (Fig. 7E). Concurrently, lactate output from aerobic glycolysis fell sharply both intracellularly and extracellularly. Mechanistically, ONOO^−^ S-nitrosylates and inactivates glyceraldehyde-3-phosphate dehydrogenase, attenuating glucose metabolism. These metabolic perturbations convert an immunosuppressive tumor microenvironment into an immunostimulatory one, characterized by M2-to-M1 macrophage repolarization, depletion of myeloid-derived suppressor cells, down-regulation of regulatory T cells, and enhanced CD8^+^ T-cell infiltration. Combined with α-PD-L1, APAP-P-NO delivered superior antitumor efficacy, offering a compelling strategy to enhance immunotherapy through targeted metabolic intervention [55]. Recently, we developed a microwave-responsive nanoplatform for hepatocellular carcinoma (HCC) therapy, which synergistically integrates microwave thermal ablation with tumor-microenvironment-triggered drug release and therapeutic gas (NO) generation to improve therapeutic efficacy (Fig. 7F) [101]. Our findings indicate that the nanomedicine not only produces NO but also potentiates the cytotoxic activity of cisplatin, leading to the accumulation of lipid peroxides and the subsequent induction of ferroptosis in HCC cells. By modulating oxidative stress through NO release, this nanodrug markedly increases the sensitivity of HCC to microwave-induced thermochemotherapy and significantly enhances therapeutic efficacy.

Different types of ROS, including O_2_·^-^, H_2_O_2_, ·OH, and ^1^O_2_, exhibit distinct biological properties and therapeutic potentials in cancer treatment. O_2_·^-^, as a primary ROS, plays a crucial role in initiating redox reactions but is relatively unstable and often serves as a precursor to other ROS. H_2_O_2_ is more stable and diffusible, acting as a key intermediate in ROS cascades and cellular signaling, although its direct cytotoxicity is limited. In contrast, ·OH possess extremely high oxidative reactivity, enabling irreversible damage to biomolecules and serving as the primary cytotoxic species in CDT, ferroptosis and pyroptosis induction, but their ultrashort lifetime restricts their action to localized regions. ^1^O_2_, the dominant effector in PDT, offers high selectivity and controllability. However, its generation is oxygen-dependent and limited by shallow tissue penetration. Overall, the distinct reactivity, stability, and diffusion characteristics of different ROS highlight the importance of developing multi-ROS synergistic or cascade amplification strategies, particularly through nanotechnology, to overcome the limitations of single ROS-based therapies and improve therapeutic efficacy and precision.

## 3 Disrupting the antioxidant defense system

To counterbalance these diverse sources of ROS and prevent oxidative damage, cells have evolved a multilayered antioxidant defense network that tightly regulates intracellular redox homeostasis. This system primarily consists of enzymatic antioxidants, including SOD, CAT, coenzyme Q(CoQ), and GPX, along with redox-regulating molecules including GSH and Trx [14]. Among these pathways, the GSH-dependent antioxidant system plays a central role in maintaining cellular redox balance. Briefly, cystine is imported from the extracellular space via system Xc^−^ (SLC3A2/SLC7A11) in exchange for intracellular glutamate. Then it is reduced to cysteine and utilized by glutathione synthetase (GS) to synthesize GSH [102]. GPX4 subsequently employs GSH to reduce phospholipid hydroperoxides (PLOOH) to PLOH, thereby suppressing lipid peroxidation and ferroptosis [103]. In parallel, mitochondrial GPX4-GSH and DHODH pathways, together with plasma membrane-associated FSP1, maintain reduced coenzyme Q_10_ (CoQ10), which functions as a radical-trapping antioxidant to inhibit oxidative membrane damage [104]. In addition, dietary antioxidants such as vitamins C and E, carotenoids, and flavonoids, along with repair enzymes responsible for eliminating oxidatively damaged biomolecules, collectively contribute to maintaining intracellular redox equilibrium [105].

Nanomedicine provides unique advantages for selectively targeting antioxidant pathways within tumors, including enhanced tumor accumulation, stimulus-responsive activation, and the capacity to integrate multiple redox-regulatory functions. Representative strategies include depletion of key reducing equivalents such as NADPH and GSH, inhibition of GSH biosynthesis through blockade of γ-glutamyl-cysteine synthetase or system Xc^−^-mediated cystine uptake, and direct suppression of antioxidant enzymes including GPX4, DHODH, and thioredoxin reductase (TrxR) [[27], [28], [29]]. Beyond single-pathway inhibition, numerous nanoplatforms have recently been engineered to simultaneously consume intracellular GSH, inhibit antioxidant enzymes, and amplify ROS accumulation, thereby efficiently collapsing tumor antioxidant defenses and sensitizing cancer cells to oxidative damage. By exploiting the intrinsic redox vulnerabilities and unique microenvironment of malignant cells, these nanotherapeutic strategies provide promising opportunities for redox-based tumor therapy (Table 2).

**Table 2:** Summary of nanotherapeutic strategies for disrupting the antioxidant defense system.

Target	Intervention strategies	Representative examples	Advantages	Limitations	Ref.
NADPH	NADPH inhibition	PMVL	High sensitivity to ferroptosis	Unexpected catalysis; Nonspecific deposition	[106]
		PCFP	NOX-like enzyme activity; High tumor selectivity; Reduced systemic toxicity	Potential metal toxicity; Immunogenicity	[107]
	NADPH depletion	L-Arg CeO2	Enhance catalytic efficiency	Limited stability	[108]
GSH	GSH-responsive delivery	PEG-PUTeTe-PEG	Ultrasensitive GSH-responsiveness; High tumor selectivity	Potential Te-related toxicity	[109]
		^DA^TAT-NP_VT_	Cascade activation strategy; Deep tissue penetration	Structural complexity; X-ray-induced toxicity	[110]
	Disrupting GSH synthesis	BZAMH NPs	Synergistic ferroptosis induction	Potential metal-related toxicity	[111]
	GSH consumption	Cu^2+^-g-C_3_N_4_	Multi-ROS induction; Photostability	Light dependence; Low quantum yield	[112]
		Mn(III)-HFs	GSH-responsive degradation; MRI guidance	Limited SDT efficiency	[113]
GPX	GPX4 inhibition	RSL3/ML162/ML210-based nanomedicines	Efficient GPX4 inhibition	Poor stability; Limited translational potential	[114,115]
		Cu-TCPP(Fe)	Synergistic inhibition; Multienzyme-like activity	Low specificity	[116]
		RSV-NPs@RBCm	Biocompatibility; Reduced systemic toxicity	Complex preparation; Limited payload capacity	[117]
	GPX1 suppression	ABFP NPs	Endogenous activation; Disrupting metabolic homeostasis	Metabolic dependence; Narrow therapeutic window	[118]
		MSA	Simple preparation	Limited efficacy	[[119], [120], [121]]
DHODH	DHODH degradation	BPNpro	Synergistic inhibition of GPX and DHODH	PROTAC dependency	[122]
	DHODH inhibition	siR/IONs@LDH	Enhances susceptibility to ferroptosis	Limited stability	[123]
Others	CoQ inhibition	iF-CuS-M/SSO@Gel	Lipid metabolism; Modulation in tumor and immune cells	Restricted to primary tumor treatment	[124]
	Trx regulation	Phy@HES-IR	Dual antioxidant blockade (GSH and Trx systems)	Potential toxicity; Oxygen-dependence	[125]
	HO-1 blockade	ZnPP	PDT enhancement; Ferroptosis promotion	Challenges in multimodal coordination	[126]

### 3.1 Inhibition or depletion of NADPH

NADPH (nicotinamide adenine dinucleotide phosphate, reduced form) is an essential intracellular electron donor, providing reducing power necessary for anabolic reactions and maintaining redox balance [127]. It exists in both cytosolic and mitochondrial pools, often in a protein-bound form, with levels varying across tissues. NADPH, as a key electron donor, sustains cellular redox homeostasis by supporting GSH regeneration and fueling the peroxiredoxin (Prx) and thioredoxin systems, which collectively contribute to ROS detoxification [128]. Given its multifaceted roles, NADPH has emerged as a promising therapeutic target in cancer. Current strategies mainly aim to restrict NADPH availability by inhibiting NADPH generation and promoting NADPH depletion.

NAD(H) and NADP(H) are structurally related cofactors that shuttle and store reducing equivalents in cells [129]. NAD^+^ and its reduced form, NADH, interconvert by accepting or donating a hydride ion (H^−^). As a pivotal metabolite in cellular homeostasis, NAD^+^ functions as a key redox cofactor in energy-generating pathways such as the Krebs cycle, fatty-acid β-oxidation, glycolysis, and serine biosynthesis [[130], [131], [132]]. The ribose ring of the adenosine monophosphate (AMP) moiety can be phosphorylated by NAD kinase (NADK) to yield NADP^+^, which is subsequently reduced to NADPH (Fig. 3A) [133,134]. In this pathway, nicotinamide phosphoribosyltransferase (NAMPT) is the rate-limiting enzyme, and numerous NAMPT-selective inhibitors (e.g., FK866, CHS828, and OT-82) have been developed, with several currently in clinical trials [[135], [136], [137], [138], [139]]. In addition, the pentose phosphate pathway (PPP) is one of the primary sources of cellular NADPH, with glucose-6-phosphate dehydrogenase (G6PD) acting as the rate-limiting enzyme and major source of cytoplasmic NADPH [140]. G6PD plays a critical role in maintaining redox homeostasis and regulating diverse biological functions [141]. Consequently, inhibiting G6PD to block NADPH production represents an effective strategy for enhancing the sensitivity of tumor cells to oxidative stress.

**Fig. 8:**
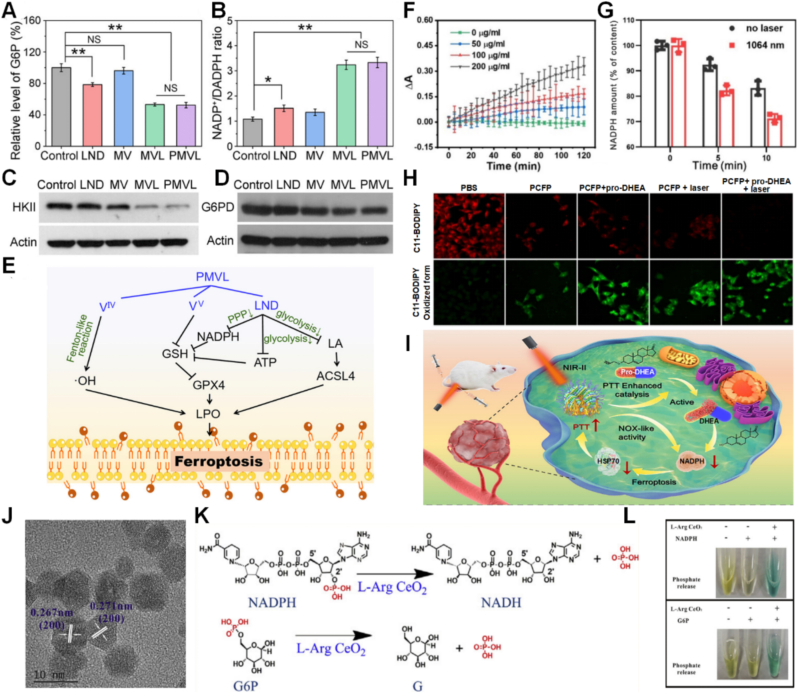
Disruption of NADPH-dependent antioxidant pathways based on nanomedicines. PMVL-mediated intervention in generation of G6P **A)** and NADPH **B)** via PPP Suppression. Measurement of HKII **C)** and G6PD **D)** expression by WB analysis in B16F10 cells after different treatments. **E)** Schematic illustration of PMVL-induced ferroptosis by suppressing NADPH generation and triggering LPO accumulation. **F)** Characterization of the NOX-like activity of PCFP by UV-Vis spectroscopy. **G)** The NADPH consumption capacity of PCFP at different time points after different treatments. **H)** C11-BODIPY 581/591 probe staining for lipid peroxidation detection to various treatments. **I)** Therapeutic Mechanisms of PCFP-mediated ferroptosis through inhibiting the production of NADPH and promoting the generation of free radicals. **J)** HRTEM image of L-Arg CeO2. **K)** The proposed dephosphorylation reaction of NADPH and G6P catalyzed by L-Arg CeO_2_. **L)** Detection of the L-Arg CeO_2_-catalyzed phosphate release from NADPH and G6P by malachite green assay. **A-E)** Reproduced with permission [106]. Copyright 2023, ACS Publications. **F-I)** Reproduced with permission [107]. Copyright 2024, ACS Publications. **J-L)** Reproduced with permission [108]. Copyright 2022, Elsevier Ltd. (For interpretation of the references to color in this figure legend, the reader is referred to the Web version of this article.)

Lonidamine (LND) and dehydroepiandrosterone (DHEA) are widely utilized G6PD antagonists. One study developed a ferroptosis-inducing nanosystem (PMVL) based on a polyphenol-vanadium oxide network. The LND payload carried by this system suppresses hexokinase II (HKII) and G6PD expression, thereby blocking PPP, disrupting NADPH generation, and enhancing tumor cell sensitivity to oxidative stress and ferroptosis (Fig. 8A–E) [106]. However, as NADPH metabolism is ubiquitous in both normal and tumor cells, systemic inhibition may cause toxicity to non-tumor tissues, limiting the clinical applicability of NADPH inhibitors. To address this challenge, a PEGylated PdCuFe (PCFP) nanozyme was designed to enable tumor-specific activation of a G6PD inhibitor prodrug (pro-DHEA) under near-infrared II (NIR-II) irradiation, restricting DHEA release to the tumor site. This approach effectively disrupts NADPH homeostasis in tumor cells, amplifies ferroptosis, and minimizes off-target toxicity (Fig. 8F–I). Such nano-prodrug strategies offers novel insights for therapeutic interventions predicated on the modulation of NADPH metabolism [107].

Another alternative strategy involves depleting intracellular NADPH by promoting its dephosphorylation. Xiong et al. developed a straightforward route to ultra-small truncated-octahedral ceria nanoparticles functionalized with L-arginine (L-Arg ceria; diameter <10 nm) (Fig. 8J) [108]. These phosphatase-mimicking nanozymes dephosphorylate NADP(H) and promote G6PD hydrolysis, thereby limiting NADPH regeneration through two pathways (Fig. 8K). In the malachite-green assay, L-Arg CeO_2_ released phosphate from NADPH and G6P and reduced the dephosphorylation activation energy, confirming its phosphatase-like activity (Fig. 8L).

Overall, NADPH-targeting strategies represent a promising approach for redox-based tumor therapy because they simultaneously impair antioxidant defenses and disrupt the metabolic demands required for rapid tumor proliferation. However, given the ubiquitous role of NADPH metabolism in normal tissues, nonspecific inhibition may result in systemic toxicity and limited therapeutic selectivity. Nanomedicine-based platforms provide important advantages by enabling tumor-targeted delivery, stimulus-responsive activation, and controlled release of NADPH-regulating agents, thereby improving therapeutic precision while minimizing off-target effects [142]. Consequently, the integration of NADPH-modulating strategies with advanced nanomaterials offers substantial potential for enhancing oxidative stress-mediated tumor therapy and promoting the clinical translation of redox-targeted therapeutics.

### 3.2 Biosynthesis suppression and depletion of GSH

**Fig. 9:**
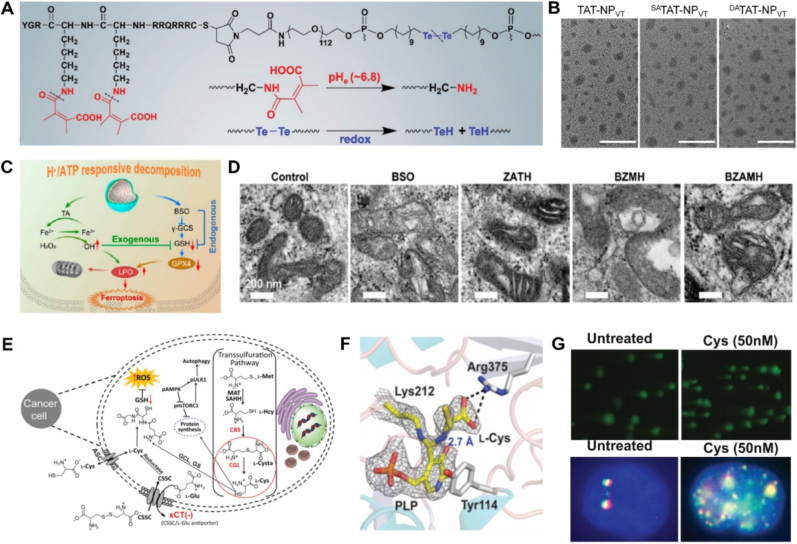
GSH-responsive nanomaterials and reduction of intracellular GSH in tumor cells. **A)** Schematic illustration of ^DA^TAT-NP_VT_ for cascaded drug delivery by extracellular acidity-promoted cellular uptake and redox-activated TPZ release. **B)** Morphology of TAT-NP_VT_, ^SA^TAT-NP_VT_, and ^DA^TAT-NP_VT_. **C)** The proposed mechanism for BZAMH NPs induced ferroptosis in tumor cells. **D)** TEM results of 4T1 cells showing the morphological changes of mitochondria. **E)** Schematic of the therapeutic effect of Cyst(e)inase treatment on cancer cells. **F)** Structural analysis of Cyst(e)inase reveals the formation of a geminal diamine reaction intermediate with substrate L-Cys. **G)** Representative images of HMVP2 PCa cells treated with vehicle or Cyst(e)inase (50 nM), along with representative images showing intracellular γH2AX and 53BP1 co-localization. **A-B)** Reproduced with permission [110]. Copyright 2022, The Royal Society of Chemistry. **C-D)** Reproduced with permission [111]. Copyright 2023, ACS Publications. **E-F)** Reproduced with permission [146]. Copyright 2017, Springer Nature. **G)** Reproduced with permission [147]. Copyright 2023, Springer Nature.

GSH, a tripeptide composed of L-cysteine, L-glutamate, and glycine, is a critical endogenous antioxidant that maintains intracellular redox homeostasis [143]. GSH exists in two redox forms, oxidized GSH (GSSG) and reduced GSH, with the latter predominating intracellularly and being continually regenerated from GSSG through NADPH-dependent processes. In tumor cells, persistent oxidative stress and elevated ROS levels often drive compensatory upregulation of GSH biosynthesis to sustain redox adaptation and therapeutic resistance [144]. This disparity has been harnessed to design GSH-responsive nanoparticles that exploit thiol-disulfide, diselenide, or ditelluride exchange reactions [145]. Y. Wang et al. engineered ditelluride-containing poly(ether-urethane) nanoparticles (PEG-PUTeTe-PEG) that release doxorubicin (DOX) upon intracellular GSH-mediated cleavage of ditelluride bonds [109]. Subsequently, Zhang et al. developed a platform (^DA^TAT-NP_VT_) that couples tumor-pH-activated TAT targeting with redox-triggered tirapazamine (TPZ) release (Fig. 9A and B) [110]. This nanoplatform utilizes elevated intracellular GSH to cleave ditelluride bonds, triggering cascade degradation and the release of hypoxia-activated TPZ, which subsequently generates cytotoxic radicals to eliminate hypoxic tumor cells. This multistage-responsive system enables precise and efficient synergistic antitumor therapy.

Another effective strategy for disrupting the GSH antioxidant system involves two complementary approaches: suppressing GSH biosynthesis and promoting intracellular GSH consumption. One representative strategy involves inhibition of γ-glutamylcysteine synthetase using agents such as L-buthionine sulfoximine (L-BSO), thereby impairing the ability of tumor cells to regenerate GSH [148,149]. Zeng et al. engineered a tumor-microenvironment-degradable hybrid nanoparticle (BZAMH NPs) to potentiate radiotherapy (RT) against triple-negative breast cancer (TNBC) [111]. Encapsulated L-BSO suppresses GSH generation, inactivating GPX4 and dismantling ferroptosis defenses, whereas co-released ferrous ions catalyze Fenton reactions that induce pronounced ferroptotic death (Fig. 9C and D). Earlier, Saha and co-workers demonstrated that combining Cyst(e)inase with L-BSO synergistically elevates ROS and induces tumor cell death [146]. Cyst(e)inase is a mutated form of CGL (CGL-E59T-E339V) that accelerates degradation of cysteine and cystine, causing profound depletion of intracellular cysteine and suppression of GSH synthesis, thereby crippling the antioxidant network, and triggering tumor cell death (Fig. 9E–F). Subsequent studies showed that Cyst(e)inase markedly increases ROS across multiple cancer cell lines, including prostate cancer (PCa), and further induces oxidative DNA damage, resulting in DNA strand breaks (Fig. 9G) [147,150].

Another complementary strategy focuses on promoting intracellular GSH consumption to weaken the antioxidant defense capacity of tumor cells and amplify oxidative stress. In particular, redox-active metal ions, including Mn, Co, Cu, and Mo, can directly react with and oxidize GSH, thereby efficiently depleting intracellular antioxidant reserves [43,112,113,151]. This process not only disrupts redox homeostasis but also facilitates subsequent ROS generation through metal ion redox cycling. For example, Ju et al. anchored Cu^2+^ onto graphitic carbon nitride nanosheets (Cu^2+^-g-C_3_N_4_); intracellularly, Cu^2+^ is reduced to Cu^+^, thereby depleting GSH and amplifying ROS-mediated oxidative stress, which enhances antitumor efficacy [112]. Similarly, Geng et al. constructed Mn^3+^-hematoporphyrin monomethyl-ether frameworks (Mn(III)-HFs) by coordinating biocompatible hematoporphyrin monomethyl ether (HMME) with Mn^3+^ [113]. Mn^3+^-HFs react with GSH to yield Mn^2+^ and GSSG, markedly reducing GSH while intensifying ROS-driven SDT.

It should be noted that GSH is ubiquitously distributed in normal tissues and participates in multiple physiological processes; therefore, nonspecific GSH depletion may induce oxidative damage and systemic toxicity. In addition, tumor heterogeneity and adaptive metabolic reprogramming may compensate for GSH depletion through alternative antioxidant pathways, thereby limiting therapeutic efficacy and durability. Consequently, future studies should focus on developing tumor-microenvironment-responsive nanoplatforms capable of achieving selective and controllable GSH depletion while minimizing off-target effects. Moreover, because the cysteine/GSH/GPX4 axis serves as a central defense mechanism against ferroptosis, intracellular GSH depletion can effectively promote lipid peroxidation and ferroptosis, providing substantial opportunities for synergistic redox-based tumor therapy.

### 3.3 Activity inhibition of GPX family

The GPX family forms a cornerstone of the cellular antioxidant defense network, metabolizing intracellular ROS and preserving redox homeostasis. GPX enzymes modulate diverse biological processes, including cell-cycle progression and signal transduction [152]. They catalyze the reduction of H_2_O_2_ and other organic peroxides to H_2_O or the corresponding alcohols, employing reduced GSH as the electron donor; this reaction safeguards biomolecules from oxidative injury and supports normal physiology and healthy aging [153]. Eight GPX isoenzymes have been identified. Five GPX isoforms (GPX1-4 and GPX6) harbor the selenocysteine-containing active site characteristic of Sec-GPX, whereas GPX5, GPX7, and GPX8 utilize cysteine in place of selenocysteine (Cys-GPX). Among these, cytosolic GPX1 and membrane-associated GPX4 are the most extensively investigated owing to their clinical importance. Aberrant expression of specific GPX isoforms has been documented in lung, esophageal, colorectal, and liver cancers, implicating these enzymes in tumor initiation, progression, and therapeutic response.

Selenoprotein phospholipid hydroperoxide glutathione peroxidase (PHGPX, GPX4) is the principal enzyme that reduces lipid hydroperoxides, with GSH serving as the reductant. By limiting membrane lipid peroxidation, GPX4 suppresses ferroptosis [154,155]. Ferroptotic cell death has emerged as a compelling anticancer strategy and is regulated chiefly by the GSH-GPX4 axis [156,157]. Depletion of GSH disables GPX4, triggers unchecked lipid peroxidation, elevates ROS, and culminates in ferroptosis [158,159]. Accordingly, GPX4 has become an attractive molecular target for precision tumor therapy. Interest in GPX4 inhibition has risen substantially in recent years. Class II ferroptosis inducers (FINs), such as RSL3, ML162, and ML210, act by inhibiting GPX4 activity. RSL3 and ML162 covalently bind the catalytic selenocysteine residue of GPX4, directly blocking its enzymatic function [114]. ML210, a nitroisoxazole compound, is converted intracellularly to the α-nitroketoxime (JKE-1674), which generates an electrophilic nitrile oxide that selectively and covalently inhibits GPX4 [115].

**Fig. 10:**
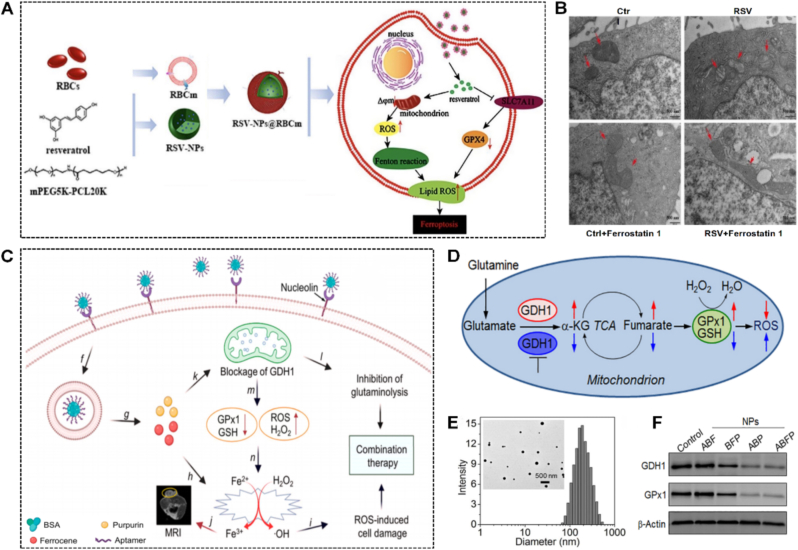
Reduce the expression or downregulate proteins related to the GPX family. **A)** The synthesis procedure of the RSV-NPs@RBCm and the mechanism of cancer treatment by ferroptosis. **B)** Characterization of ferroptosis by TEM in HT29 cells treated with RSV or Ferrostatin-1. **C)** Schematic illustration of the tumor-targeted theranostic NP platform for effective BCa therapy via a combination of mitochondrial glutaminolysis inhibition and CDT. **D)** Schematic illustration of GDH1-mediated glutamine metabolism in the mitochondria to regulate GPX1 expression and ROS generation in tumor cells. **E)** Size distribution and morphology of ABFP NPs. **F)** Western blot analysis of GDH1 and GPX1 expression in MCF-7 cells after 12 h of culture with different materials. **A-B)** Reproduced with permission [117]. Copyright 2022, Elsevier Ltd. **C-F)** Reproduced with permission [118]. Copyright 2021, ACS Publications.

Li et al. designed a copper-porphyrin metal-organic framework (MOF) system, specifically Cu-TCPP(Fe), that integrates gold nanoparticles with RSL3 [116]. The framework displays enzyme-like activity, broadly suppressing anti-ferroptotic pathways in tumor cells and thereby intensifying ferroptosis. Within acidic lysosomes, the nanosheets release supramolecularly bound RSL3, which then targets the selenocysteine residue of GPX4 and further diminishes its anti-ferroptotic capacity. Similarly, Zhang et al. developed a biomimetic nanocarrier by coating poly(ε-caprolactone)/poly(ethylene glycol) (PCL/PEG) nanoparticles with red-blood-cell membranes (RSV-NPs@RBCm) (Fig. 10A) [117]. Resveratrol (RSV), a bioactive polyphenolic monomer from traditional Chinese medicine, exerts therapeutic effects via multiple targets and mechanisms [160]. Cells treated with RSV or its formulations show pronounced reductions in mitochondrial size and cristae density (Fig. 10B).

GPX1, a homotetramer and the first identified selenoprotein, is overexpressed in many tumors, where it efficiently scavenges ROS, limits apoptosis, confers drug resistance, and promotes cancer-cell survival [161,162]. In cisplatin-resistant non-small-cell lung-cancer cell lines, GPX1 expression is markedly elevated; its overexpression increases cisplatin resistance, whereas knockdown restores cisplatin sensitivity [163]. Accordingly, recent efforts have centered on down-regulating GPX1 expression or activity to suppress tumor growth. Xu et al. engineered a tumor-targeted nanoparticle platform composed of bovine serum albumin (BSA), ferrocene, and purpurin (ABFP NPs) (Fig. 10C–E) [118]. Purpurin, an FDA-approved natural compound, potently inhibits glutamate dehydrogenase 1 (GDH1), a mitochondrial enzyme overexpressed in breast, liver, and lung cancers (Fig. 10D) [164]. GDH1 blockade disrupts mitochondrial glutaminolysis and perturbs cellular redox balance, thereby lowering GPX1 and GSH levels, elevating ROS, and ultimately suppressing tumor proliferation (Fig. 10F). Small-molecule GPX1 inhibitors have also been explored. Mercaptosuccinic acid (MSA) is the classical competitive inhibitor that occupies the selenocysteine active site of GPX1 [119]. Pentathiepins, a newly identified inhibitor class, exhibit substantially stronger potency than MSA. Combination regimens that pair GPX1 inhibitors with PDT have been shown to intensify oxidative stress, increase intracellular ROS, promote tumor-cell apoptosis, and yield synergistic antineoplastic effects [120,121]. Conversely, elevated GPX1 activity may underlie resistance to conventional chemotherapy: lymphoma cells isolated from patients refractory to methotrexate and etoposide display significantly up-regulated GPX1 expression.

Beyond GPX1 and GPX4, increasing evidence indicates that other GPX family members also participate in tumor initiation, progression, metastasis, and therapeutic resistance through regulation of intracellular redox homeostasis. For instance, GPX2 is frequently upregulated in esophageal, colorectal, prostate, and head-and-neck cancers, and its elevated expression is often associated with poor prognosis and enhanced tumor survival under oxidative stress conditions [165,166]. GPX3, the only extracellular selenoprotein within the GPX family, scavenges extracellular ROS and modulates the tumor microenvironment. Notably, GPX3 exhibits context-dependent functions in cancer, acting either as a tumor suppressor or as a pro-survival factor depending on tumor type and redox status. Aberrant GPX3 expression has been reported in multiple gastrointestinal malignancies [167]. In gastric cancer, hypermethylation of the GPX3 promoter is associated with lymph-node metastasis, whereas GPX3 downregulation in ovarian cancer suppresses clonogenic growth and cell viability [168]. Emerging studies have further implicated GPX5-GPX8 in tumor-associated redox regulation; however, their precise biological functions and therapeutic relevance remain incompletely understood. Collectively, these findings highlight the functional heterogeneity of the GPX family in cancer biology and underscore the importance of context-dependent redox regulation in determining tumor progression and therapeutic response. Future studies should therefore focus on clarifying the distinct functions, regulatory mechanisms, and tumor-specific dependencies of individual GPX members, while developing selective inhibitors and nanomedicine-based delivery systems to improve therapeutic specificity and minimize systemic toxicity.

### 3.4 DHODH inhibition

DHODH is an iron-containing flavin-dependent mitochondrial enzyme that catalyzes the fourth step of de novo pyrimidine biosynthesis, thereby underpinning cellular metabolism and proliferation [169,170]. As an iron-sulfur flavoprotein, DHODH collaborates with mitochondrial GPX4 but acts independently of cytosolic GPX4 or FSP1 to restrain ferroptosis by reducing extramitochondrial CoQ_10_ and vitamin K [104,171]. Owing to this pivotal role in controlling lipid-peroxidation, DHODH has become an attractive anticancer target. Inhibitors such as brequinar selectively impeded the growth of GPX4-deficient tumors by provoking ferroptosis [104,172,173]. Because DHODH and mitochondrial GPX4 form the two principal mitochondrial defenses against lipid peroxidation, blocking one pathway compels tumor cells to depend on the other; simultaneous inhibition of both, therefore, amplifies ferroptosis and enhances antitumor efficacy.

**Fig. 11:**
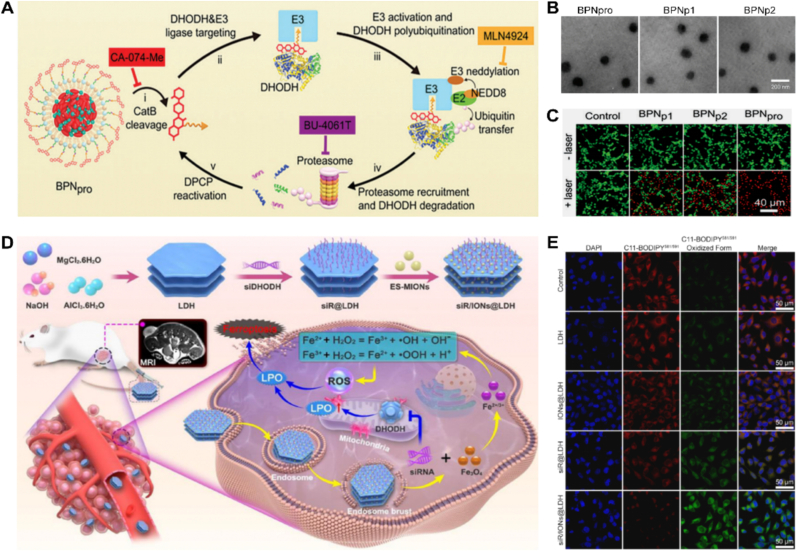
Induce DHODH degradation or inhibit DHODH expression. **A)** Proposed mechanism of DHODH degradation induced by DPCP after CatB activation. **B)** TEM images of BPNpro, BPNp1, and BPNp2. **C)** PBS, BPNp1, BPNp2, and BPNpro were incubated for 24 h, and the cells were seen alive and dead after light exposure. **D)** schematic illustration of the construction and theranostic mechanism of the siR/IONs@LDH nanoplatform. **E)** Fluorescence microscope images of 4T1 cells with various treatments for 4 h and subsequently stained with C11-BODIPY581/591 to determine the lipid peroxidation. **A-C)** Reproduced with permission [122]. Copyright 2023, John Wiley & Sons. **D-E)** Reproduced with permission [123]. Copyright 2023, ACS Publications.

Proteolysis-targeting chimera (PROTAC), the cornerstones of targeted-protein-degradation therapy, have attracted considerable attention since their inception over two decades ago [174]. PROTAC are bifunctional small molecules composed of two ligands joined by a linker: one engages the protein of interest (POI), whereas the other recruits an E3 ubiquitin ligase. Assembly of a ternary POI-PROTAC-E3 complex triggers POI ubiquitination and subsequent degradation via the ubiquitin-proteasome system (UPS), after which the PROTAC is released to catalyze further degradation cycles [175]. Yao et al. introduced a multifunctional nanoagent (BPNpro) that integrates a GPX4-targeting boron-dipyrromethene (Bodipy) probe (BP) with a DHODH-directed PROTAC [122]. By concurrently disabling GPX4 and DHODH, BPNpro induced robust ferroptosis and tumor-cell death, representing the first demonstration of a dual-inhibition strategy. This dual-functional PROTAC is composed of a tumor-microenvironment-responsive cathepsin B (CatB)-cleavable PROTAC peptide (DPCP), an E3-ligase-targeting peptide, and a brequinar moiety that binds DHODH (Fig. 11A–C). A thermosensitive phospholipid shell encapsulates DPCP and modulates the release of the B. DPCP was cleaved by CatB, liberating its active fragments to bind DHODH and recruit the E3 ligase. The ensuing UPS-mediated DHODH degradation allowed DPCP to initiate successive rounds of target depletion. Compared with traditional small-molecule inhibitors, monoclonal antibodies, and nucleic-acid therapeutics, PROTAC confer higher pharmacological efficiency and lower dosing requirements [176].

In addition to direct DHODH degradation, inhibiting DHODH expression can potentiate ferroptosis by weakening cellular ferroptosis-defense pathways. Chen et al. designed a layered-double-hydroxide (LDH) nanoparticle platform co-loaded with iron-oxide nanoparticles (IONs) and a DHODH-targeting siRNA (siR/IONs@LDH) (Fig. 11D). Lipid peroxidation, evaluated with C11-BODIPY 581/591 staining, confirmed the superior lipid-peroxide-generating capacity of siR/IONs@LDH (Fig. 11E) [123]. Mn^2+^ ions similarly promote ROS accumulation in tumor cells, disrupt redox homeostasis, and trigger cell death [177]. Emerging evidence suggests that Mn^2+^ can further modulate ferroptosis-related pathways at the molecular level. Mn^2+^ was shown to downregulate DHODH by elevating type I interferon (IFN) production, thereby inducing ferroptosis in tumor cells [178].

As a key mitochondrial ferroptosis suppressor functioning in parallel with the GPX4-GSH and FSP1-CoQ systems, DHODH has emerged as a highly promising therapeutic target for inducing ferroptosis [179]. Increasing evidence suggests that simultaneous disruption of multiple ferroptosis-defense pathways, rather than inhibition of a single antioxidant mechanism, may achieve more sustained lipid-peroxide accumulation and more potent ferroptotic responses in tumor cells. Nevertheless, the precise interplay among DHODH, mitochondrial metabolism, pyrimidine biosynthesis, and other redox-regulatory networks remains incompletely understood. Therefore, future studies should focus on developing tumor-selective and molecularly precise nanoplatforms capable of controllably modulating DHODH activity while synergistically integrating ROS amplification, GPX4 inhibition, and ferroptosis induction. Such multimodal redox-regulatory strategies may provide new opportunities for overcoming tumor antioxidant adaptation and improving the therapeutic efficacy of ferroptosis-based cancer treatment.

### 3.5 Others

**Fig. 12:**
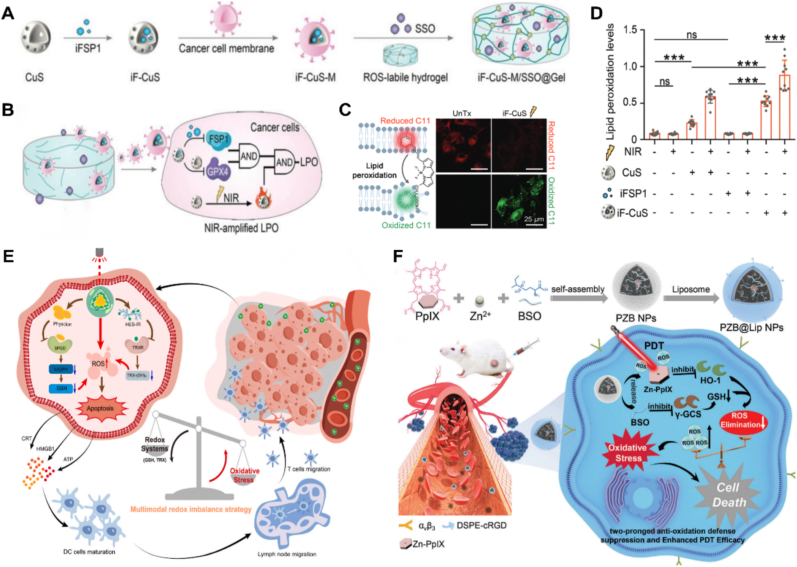
Anti-tumor nanomedicine based on FSP-1 and Trx inhibition. **A)** A scheme for the assembly of the composite hydrogel. **B)** The composite hydrogel senses ROS in the TME for the release of iF-CuS-M and SSO. The iF-CuS-M induces lipid peroxidation in cancer cells via two parallel pathways, whose effects are further amplified by NIR irradiation. **C)** Scheme for the measurement of lipid peroxidation and fluorescent images of BODIPY C11 in 4T1 cells. **D)** Relative lipid peroxidation levels quantified as the ratio of oxidized to reduced form of BODIPY C11. **E)** Schematic illustration of multistage redox regulation strategy for photodynamic immunotherapy. **F)** Schematic of the synthesis of PZB@Lip NPs and schematic showing the reversal of the tolerance for ROS in tumor cells after dual antioxidation defense suppression using PZB@Lip NPs to enhance the efficacy of PDT. **A-D)** Reproduced with permission [124]. Copyright 2023, John Wiley & Sons. **E)** Reproduced with permission [125]. Copyright 2024, Elsevier Ltd. **F)** Reproduced with permission [126]. Copyright 2021, John Wiley & Sons.

Beyond the canonical antioxidant systems, additional redox-regulating networks also contribute to the maintenance of cellular redox homeostasis. Notably, CoQ, thioredoxin TrxR, and ceruloplasmin play important roles in modulating oxidative stress through distinct mechanisms. Targeting these noncanonical antioxidant systems can likewise disrupt redox balance, promote ROS accumulation, and enhance oxidative stress-mediated antitumor effects. CoQ, also termed ubiquinone (UQ), was first isolated in 1957 by Crane and colleagues as a redox-active mitochondrial lipid [180]. Initially recognized as an essential component of the mitochondrial respiratory chain (MRC), CoQ is now known to participate in numerous cellular redox reactions [181]. Beyond its role in electron transport, CoQ scavenged lipid-peroxyl radicals, thereby limiting lipid peroxidation and ferroptosis [182]. In 2019, two groups independently identified the same CoQ oxidoreductase, later renamed FSP1, which functioned as a glutathione-independent ferroptosis inhibitor [171,183]. Situated at the plasma membrane, FSP1 reduced CoQ to CoQH_2_, which served as a radical-trapping antioxidant that prevents lipid peroxidation and thus blocked ferroptosis. Because ferroptosis represents a distinct, therapeutically exploitable mode of cell death, pharmacologically targeting the FSP1-CoQ_10_-NADPH axis emerged as a promising strategy for otherwise refractory cancers. Current FSP1 inhibitors fall into two categories. Specific inhibitors include iFSP1, Versatile Inhibitor of FSP1, Ferroptosis Sensitizer 1, and NPD4928. Non-specific inhibitors include IR1, QiLing Decoction (QLD), curcumin, and propofol [184]. Among these, iFSP1 is the most widely used: it not only induces ferroptosis by blocking FSP1 but also indirectly stimulates antitumor immune responses. Because the FSP1 pathway operates in parallel with the glutathione-dependent GPX4 pathway, simultaneous inhibition of both systems augmented lipid peroxidation. In this context, Ma et al. developed a composite hydrogel (iF-CuS-M/SSO@Gel) that embeds hollow mesoporous CuS nanoparticles, which exhibit intrinsic GPX4-inhibitory activity (Fig. 12A and B), while co-delivering iFSP1 to disable FSP1 (Fig. 12C and D). The photothermal effect of CuS further promoted immunogenic cell death (ICD), thereby maximizing lipid peroxidation and ICD induction [124].

The Trx system, comprising Trx, TrxR, and NADPH, represents another major intracellular antioxidant defense system. It counteracts oxidative stress, preserves redox homeostasis, and modulates cell-survival signaling [185]. Trx, a ubiquitous and highly conserved protein, modulates the cellular redox state by reducing oxidized proteins. Intracellularly, Trx cycles between its reduced form, Trx-(SH)_2_, and the oxidized Trx-S_2_, to exert its antioxidant function. Reduced Trx directly neutralizes ROS such as H_2_O_2_ and ·OH. Once oxidized to Trx-S_2_, it temporarily loses activity until TrxR, powered by NADPH, reduces the disulfide bond and restores Trx activity [186]. Trx has therefore become a key biomarker for disorders associated with oxidative stress, inflammation, and aging [187]. Tumors exploit the Trx pathway to buffer metabolism-driven ROS, sustain proliferation and angiogenesis, and optimize nutrient and oxygen supply; accordingly, the system represents an attractive therapeutic target [188]. Xiong et al. synthesized a hydroxyethyl-starch nanomedicine (Phy@HES-IR) that leverages Trx biology (Fig. 12E). They discovered that IR820, in addition to acting as a photosensitizer for PDT, inhibited TrxR, thereby blocking the Trx antioxidant route and intensifying oxidative stress within tumor cells. Phy@HES-IR also interfered with the pentose-phosphate pathway, suppressing intracellular GSH biosynthesis. Concurrent inhibition of the GSH and Trx defenses markedly enhanced PDT efficacy, induced ICD, and amplified the antitumor immune response [125].

Ceruloplasmin (Cp), also called ferroxidase, and heme oxygenase-1 (HO-1) are integral components of the cellular antioxidant system that safeguard against oxidative stress. Evidence indicates that Cp drives tumor progression mainly by modulating ferroptosis, promoting angiogenesis, and reshaping the tumor microenvironment. Acting as a copper-ion carrier, Cp distributes Cu^2+^ to pro-angiogenic pathways, thereby facilitating neovascularization [189]. Cp also catalyzes iron oxidation: its copper centers transfer electrons to O_2_, converting Fe^2+^ to Fe^3+^; the resulting Fe^3+^ then binds transferrin (TF) and is imported to satisfy the heightened iron demand of proliferating cells. In lung, liver, and melanoma cancer cells, Cp up-regulation accelerates the Fe^2+^ to Fe^3+^ conversion, depletes the intracellular Fe^2+^ pool, and suppresses ferroptosis [190]. Consequently, elevated Cp expression can curtail ferroptotic cell death and promote tumor progression. Although nanomaterial-based targeting of Cp remains largely unexplored, focusing on Cp may open new avenues for ferroptosis-centered anticancer therapy.

HO-1 is a metabolic enzyme with potent antioxidant capacity. By degrading heme into carbon monoxide, biliverdin, and iron, HO-1 suppresses inflammation and apoptosis, making it a pivotal cellular antioxidant [191]. HO-1 modulates breast-cancer cell proliferation, invasion, metastasis, angiogenesis, the immune microenvironment, and therapy resistance [192,193]. To undermine these redox defenses, researchers have combined HO-1 blockade with other antioxidant-pathway inhibitors. A notable example is the multifunctional nanomedicine PZB NP**s** (Fig. 12F), which co-encapsulate the GSH inhibitor BSO and the HO-1 inhibitor zinc protoporphyrin (ZnPP). This formulation simultaneously disabled tumor-cell antioxidant defenses and markedly enhanced photodynamic-therapy efficacy [126]. Additional antioxidant enzymes, including SOD, POD, and CAT, play crucial roles in redox regulation. SOD converts O_2_·^-^ to H_2_O_2_, its inhibition can, therefore, heighten oxidative stress. PODs decomposed organic peroxides, often acting in concert with CAT, which further broke down H_2_O_2_ into H_2_O and O_2_. Selective CAT inhibition (to accumulate H_2_O_2_) or SOD up-regulation (to raise intracellular peroxide levels) can intensify oxidative damage and bolster antitumor responses.

In summary, tumor cells rely on highly interconnected antioxidant systems to maintain redox homeostasis and adapt to persistent oxidative stress. Importantly, these antioxidant pathways exhibit substantial functional redundancy and compensatory crosstalk; consequently, inhibition of a single pathway is often insufficient to induce sustained oxidative stress or durable ferroptotic responses, as tumor cells can activate alternative redox-buffering mechanisms to preserve survival. Moreover, the metabolic plasticity of the tumor cells further complicate effective disruption of antioxidant defenses. Therefore, future studies should focus on elucidating the dynamic interactions among distinct antioxidant networks and developing tumor-selective, spatiotemporally controllable nanoplatforms capable of simultaneously modulating multiple redox pathways. Such integrated strategies may enable more precise and sustained disruption of tumor redox homeostasis.

## 4 Conclusions and perspectives

Targeting the profound redox dysregulation characteristic of malignant tumors has emerged as a highly promising therapeutic strategy. Nanomedicine-enabled modulation of redox homeostasis represents a paradigm shift toward precision tumor therapy. Specifically, rationally designed nanotherapeutics can synergistically amplify intracellular ROS/RNS generation while concurrently suppressing key antioxidant defense systems, thereby driving tumor cells beyond their oxidative stress tolerance threshold. In contrast to conventional small-molecule redox modulators, which are frequently limited by rapid systemic clearance, poor tumor accumulation, and dose-limiting off-target toxicity, engineered nanoplatforms such as nanozymes, single-atom catalysts, metal-organic frameworks, and stimulus-responsive nanocarriers offer substantial advantages. These advanced systems facilitate selective tumor accumulation through enhanced EPR and/or active targeting, permit spatiotemporally precise control over catalytic activity, and enable multimodal integration of complementary redox-modulating mechanisms within a single platform. Collectively, these features promote irreversible oxidative damage and orchestrate regulated cell death, with particular efficacy toward ferroptosis, apoptosis, and pyroptosis.

Encouragingly, the translational momentum of redox-targeted nanomedicine is accelerating. In addition to clinically proven PDT, several ROS-amplifying nanotherapeutic strategies have entered preclinical and early translational evaluation. For example, FDA-approved iron oxide nanoparticles such as ferumoxytol have been repurposed in clinical trials as peroxidase-mimicking nanozymes to induce ferroptosis and repolarize tumor-associated macrophages [194,195]. Similarly, hafnium oxide nanoparticles (NBTXR3/Hensify), which trigger massive localized ROS generation upon X-ray irradiation, have received clinical approval for specific sarcomas, validating the feasibility of nano-amplified oxidative stress in human patients [196,197]. These advances collectively suggest that nanomedicine is transitioning redox-based therapy from conceptual exploration toward clinically actionable intervention strategies.

Nevertheless, several critical bottlenecks continue to limit the therapeutic efficacy and clinical translation of redox-modulating nanomedicines. First, the heterogeneous tumor microenvironment, particularly severe hypoxia and insufficient endogenous substrates such as H_2_O_2_, substantially restricts the catalytic efficiency of ROS-generating systems *in vivo* [198]. Accordingly, future designs should prioritize self-supplying catalytic nanoplatforms capable of oxygen evolution, substrate amplification, or hypoxia-tolerant ROS generation, such as type-I PDT and Fenton-like reactions. Second, tumor antioxidant systems exhibit extensive compensatory redundancy, whereby inhibiting a single axis alone frequently activates alternative ferroptosis-defense pathways, thereby attenuating therapeutic efficacy. Consequently, multifunctional nanoplatforms capable of simultaneously targeting multiple antioxidant pathways may be required to achieve durable disruption of tumor redox homeostasis. Furthermore, the *in vivo* biological behavior of nanomedicines, including protein-corona formation, limited biodegradability, uncertain long-term metabolism, and off-target oxidative toxicity remains insufficiently understood. Developing biomimetic cloaking strategies and biodegradable nanodrygs with high catalytic specificity will be paramount for ensuring systemic biosafety and clinical viability.

Importantly, the future potential of redox-modulating nanomedicine extends beyond directly oxidative tumor damage. Increasing evidence demonstrates that severe oxidative stress can profoundly remodel the immunosuppressive tumor microenvironment by inducing immunogenic cell death , promoting the release of DAMPs, enhancing dendritic-cell maturation and antigen presentation, and activating cytotoxic CD8^+^ T-cell responses [199,200]. Thus, redox nanotherapeutics are emerging not only as oxidative tumoricidal agents but also as potent immunomodulatory sensitizers capable of converting immunologically “cold” tumors into “hot” inflamed phenotypes. Future therapeutic paradigms will focus on the synergistic integration of redox modulation with immune checkpoint blockade, radiotherapy, tumor ablation, catalytic gas therapy, and metabolic intervention. Moreover, a deeper mechanistic understanding of tumor-specific redox dependencies, together with the identification of predictive biomarkers for oxidative vulnerability, will be essential for patient stratification and personalized therapeutic design. Collectively, continued advances in redox biology, nanotechnology, and tumor immunology are expected to establish nanomedicine-powered redox homeostasis modulation as a highly effective and clinically translatable paradigm for precision oncology.

## CRediT authorship contribution statement

**Jiale Zhan:** Conceptualization, Data curation, Formal analysis, Investigation, Methodology, Project administration, Software, Validation, Visualization, Writing – original draft. **Ruie Chen:** Formal analysis, Methodology, Visualization, Writing – review & editing. **Muhe Chen:** Formal analysis, Writing – review & editing. **Shuyue Yan:** Data curation, Investigation, Validation, Writing – original draft. **Yitian Zhang:** Conceptualization. **Jiawen He:** Conceptualization. **Ya Meng:** Writing – review & editing. **Xiangrong Yu:** Funding acquisition, Resources, Supervision, Writing – review & editing. **Liewei Wen:** Conceptualization, Formal analysis, Funding acquisition, Resources, Supervision, Writing – review & editing.

## Declaration of competing interest

The authors declare that they have no known competing financial interests or personal relationships that could have appeared to influence the work reported in this paper.

## Data Availability

Data will be made available on request.
